# A Retrospective Claims-Based Study Evaluating Clinical and Economic Burden Among Patients With Moderate to Severe Osteoarthritis Pain in the United States

**DOI:** 10.36469/jheor.2022.31895

**Published:** 2022-03-01

**Authors:** Patricia B. Schepman, Sheena Thakkar, Rebecca L. Robinson, Craig G. Beck, Deepa Malhotra, Birol Emir, Ryan N. Hansen

**Affiliations:** 1 Pfizer Inc, New York, New York; 2 Eli Lilly and Company, Indianapolis, Indiana, USA; 3 Pfizer Ltd, Surrey, UK; 4 The CHOICE Institute, School of Pharmacy, University of Washington, Seattle, Washington, USA

**Keywords:** osteoarthritis, moderate pain, severe pain, treatment patterns, healthcare utilization costs

## Abstract

**Background:** There has been limited evaluation of medication adherence, healthcare resource utilization (HCRU), and healthcare costs over time in patients with osteoarthritis (OA), and stratification by pain severity level has not been reported. Assessing such longitudinal changes may be useful to patients and healthcare providers for tracking disease progression, informing treatment options, and employing strategies to optimize patient outcomes. **Objectives:** To characterize treatment patterns, HCRU, and costs over time in patients with moderate to severe (MTS) OA pain in the United States. **Methods:** We conducted a retrospective claims analysis, using IBM® MarketScan® databases, from 2013-2018. Eligible patients were aged ≥45 years with ≥12 months pre-index (baseline) and ≥24 months (follow-up) of continuous enrollment; index date was defined as a physician diagnosis of hip or knee OA. An algorithm was employed to identify MTS OA pain patients, who were propensity score matched with patients having non-MTS OA pain. Data were summarized using descriptive statistics and univariate analyses. **Results:** After propensity score matching, the overall OA pain cohorts consisted of 186 374 patients each: 61% were female, mean age was 63 years, and two-thirds (65.6%) were of working age (45-65 years). Sleep-related conditions, anxiety, and depression were significantly higher in the MTS OA pain cohort vs non-MTS (*P*<0.001). At baseline and 12- and 24-month follow-ups, receipt of prescription pain medications, HCRU, and direct medical costs were significantly higher in the MTS OA pain cohort (all *P*<0.01). Medication adherence was significantly higher in the MTS OA pain cohort for all medication classes except analgesics/antipyretics, which were significantly lower vs the non-MTS OA pain cohort (all *P*<0.0001). **Conclusions:** The burden of MTS OA pain is substantial, with patterns that show increasing medication use, HCRU, and costs vs non-MTS OA pain patients over time. Understanding the heterogeneity within the OA population may allow us to further appreciate the true burden of illness for patients in pain.

## BACKGROUND

Osteoarthritis (OA) is a chronic joint disease involving the cartilage and surrounding tissues that most commonly affects the hands, hips, and knees.^1^ An estimated 32.5 million adults in the United States are affected by OA, which is considered a leading cause of disability worldwide.^2–4^ Approximately 80% of patients with OA have some degree of movement limitation that impacts activities of daily living^5,6^ and results mainly from the presence of pain, which further increases the substantial physical, emotional, and societal burdens associated with OA.^1,5–14^ OA is also associated with comorbidities including anxiety, depression, cardiovascular disease, and sleep disorders.^9,15^ This higher comorbidity burden often contributes to increased pain and decreased physical function^15,16^ and appears to correlate with higher OA pain severity and use of prescription pain medications.^17–19^

Numerous guidelines have been published regarding the nonpharmacological and pharmacological management of OA. The 2019 American College of Rheumatology guidelines for management of OA emphasize optimizing benefit-risk outcomes for each patient, and recommend a multimodal approach based on efficacy, safety, tolerability, patient preference, and appropriateness of pharmacologic treatments.^20^ Recommendations for pharmacologic treatments are influenced by the affected joint(s), comorbidities, and other factors. Such treatments may include nonsteroidal anti- inflammatory drugs (NSAIDs) plus a proton pump inhibitor, as well as intra-articular (IA) corticosteroids and hyaluronic acid (HA).^20–22^ Opioids are often restricted for use after apparent failure of other analgesics and only for a short period of time, or are not recommended for use.^21,23^ Among studies that have examined real-world prescribing patterns of pain medications to treat OA,^6,18,24–28^ several have shown higher prescription pain medication use in patients with OA and increasing OA pain severity.^6,18,19^

Regardless of published treatment guidelines and available pharmacologic options for the symptomatic treatment of OA, the current standard of care may be inadequate since patients often continue to experience unsatisfactory pain levels despite treatment.^13,14,19,28^ One factor that often contributes to health outcomes is medication adherence (ie, the need for patients to follow their prescribed treatment regimens).^29–35^ Although no studies of adherence were identified in OA, studies in other rheumatologic diseases report suboptimal levels of medication adherence,^36–38^ and low adherence to all types of treatment modalities in musculoskeletal conditions has prompted specific consideration.^39^

The economic impact of OA has been shown to include both direct medical costs and indirect costs associated with patient treatment expenses as well as lost productivity.^6,7,9–12,24,40–44^ Furthermore, healthcare resource utilization (HCRU) across a variety of resouce categories, including medication, laboratory tests, inpatient and outpatient visits, and surgeries and procedures contributes substantially to the burden of OA. HCRU and the related costs appear to be associated with disease-level or OA pain severity, and studies have shown higher HCRU and costs with increasing OA pain severity.^19,45–48^

Longitudinal studies of OA-related treatment patterns have been   reported.^25–27^ However, there has been limited evaluation of medication adherence, HCRU, and healthcare costs over time in patients with OA, and stratification by pain severity level has not been reported. Assessing such longitudinal changes may be useful to patients and healthcare providers for tracking disease progression, informing treatment options, and employing strategies to optimize patient outcomes.

The aim of this study was to examine clinical comorbidities and changes in treatment patterns, medication adherence, HCRU, and healthcare costs over 12- and 24-month follow-up periods in patients with moderate to severe (MTS) OA pain, compared with a matched cohort of patients with non-MTS OA pain using a large, geographically dispersed patient population.

## METHODS

Data for this retrospective cohort study were from the IBM® MarketScan® Commercial Claims and Encounters and Medicare Supplemental and Coordination of Benefits claims databases^49^ from January 1, 2013, to December 31, 2018. These databases contain fully integrated, deidentified, patient-level health data from a large selection of employers, health plans, and government and public entities. Available data include patient demographics, medical and pharmacy claims, and direct medical costs.

This analysis included data from patients aged ≥45 years who had a diagnosis of hip OA, knee OA, or unspecified OA, plus a diagnosis of pain in the hip or knee within 3 months after the first OA diagnosis within the study period (index date), per International Statistical Classification of Diseases and Related Health Problems (ICD) diagnostic codes (**Supplemental Table 1**). Patients <45 years were excluded due to the smaller number of patients in this age cohort and the different presentation of OA in these younger population (eg, sports injuries). Patients with a diagnosis of neoplasm-related pain (ICD-9 338.3 or ICD-10 G89.3) were excluded. Patients with OA were required to have at least 3 years of continuous pharmaceutical and medical benefit enrollment, including 12 months pre-index (baseline) and 24 months post-index (follow-up).

Patients were classified as having MTS OA pain if they met at least one of the following criteria: (1) a visit to a rheumatology, orthopedic, or pain management specialist within 3 months post-index; (2) a surgical or nonsurgical invasive procedure relating to OA treatment (eg, joint replacement, osteotomy, arthroscopy, joint injection) (**Supplemental Table 1**) within the 12-month post-index period; (3) prescriptions for ≥2 different topical or oral NSAIDs within 3 months post-index; (4) prescriptions for ≥2 different opioids within 3 months post-index; or (5) an emergency room (ER) visit for hip and/or knee OA within 12 months post-index, followed by a primary care physician visit within 14 days of the ER visit. This definition came from a case-selection algorithm that was based on a targeted review of the published literature, treatment guidelines, professional organizations, and expert opinion.^50^ The ability of the algorithm to correctly identify patients with MTS OA was confirmed by patient medical record review, with high positive predictive value and specificity.^50^ A comparison cohort, non-MTS OA pain, comprised patients who did not have MTS OA pain.

To minimize selection bias, the patients with MTS OA pain were propensity score–matched (PSM) 1:1 with the non-MTS OA pain cohort on baseline age; gender; health plan type; region; obesity; anxiety; depression; Charlson Comorbidity Index (CCI)^51^; and CCI-specific conditions: chronic pulmonary disease, rheumatologic disease, diabetes with chronic complications, and any malignancy including leukemia and lymphoma. The CCI and CCI-specific conditions chosen in the PSM, reflective of mortality risk, are important components of outcomes studies that use administrative health data.^52^ Matches were based on the rule of <10% in the standardized difference. Outcomes included baseline patient demographics (used for PSM) and clinical characteristics including CCI score, CCI comorbidities, and other prespecified comorbidities of interest (eg, sleep-related conditions, obesity, anxiety, depression) and were assessed cumulatively at 12 and 24 months post-index. Definitions of all outcomes analyzed can be found in **Supplemental Table 2**.

Medication was defined at the class level using NDC codes from the claims, and medication adherence was defined as proportion of days covered (PDC) and was calculated as the number of days covered in the period by a prescription/drug class divided by the number of days in the period.^53^ PDC at 12 and 24 months was examined for patients with OA pain who initiated treatment within 30 days post-index. Patients with overlapping prescriptions for the same drug were not double counted when calculating the number of days covered. PDC was calculated for oral medications and for patients who initiated a drug of interest within 30 days after the index date.

Data were summarized for PSM cohorts using descriptive statistics, including means with standard differences for continuous variables, and counts and proportions for categorical variables. Differences in demographic and clinical characteristics were evaluated using *t* tests and chi-square tests for continuous and categorical variables, respectively. Comparisons from baseline in medication use at 12 and 24 months were conducted using chi-square tests to generate *P* values with patient-level data. Statistical significance was defined as *P*<0.05. All analyses were conducted using Statistical Analysis System® version 9.4 or higher (SAS Institute, Cary, North Carolina).

## RESULTS

### Demographics and Clinical Characteristics

From January 2013 to December 2018, there were 91 019 047 patients enrolled in the IBM® MarketScan® Commercial Claims and Encounters and MDCR claims databases (**Supplemental Figure 1**). Among these patients, 546 254 met the inclusion criteria, of whom 342 019 (62.6%) were identified as having MTS OA pain and 204 235 (37.3%) were identified having non-MTS OA pain. PSM resulted in 186 374 patients in each cohort and, as shown in Table 1, the cohorts had a mean age of 63 years, 66% were between 45-65 years of age, and the majority (61%) were female. Approximately two- thirds (63%) of the cohorts were enrolled in commercial plans, with the remaining patients in Medicare Supplemental plans: the majority of patients were enrolled in Preferred Provider Organization plans (PPO; 51%), followed by Comprehensive plans (20%) and Health Maintenance Organization plans (HMO; 11%). Geographic representation was predominantly in the South (35%) and North Central (30%) regions of the United States.

**Table 1.** Patient Demographics and Clinical Characteristics for Propensity Score–Matched Cohorts^a^

**Variable**	**MTS**	**Non-MTS**	**Std Diff**
Mean age (SD)	62.7 (11.2)	62.69 (11.2)	0.00
Age group, n (%)			0.00
45-65 years old	122 315 (65.6)	122 315 (65.6)	
> 65 years old	64 059 (34.4)	64 059 (34.4)	
Female, n (%)	113 750 (61.0)	113 750 (61.0)	0.00
Insurance			0.00
CCAE	118 067 (63.4)	118 067 (63.4)	
MDCR	68 307 (36.7)	68 307 (36.7)	
Type of health plan, n (%)^b^			0.00
PPO	94 973 (51.0)	94 973 (51.0)	
Comprehensive	38 098 (20.4)	38 098 (20.4)	
HMO	20 049 (10.8)	20 049 (10.8)	
Geographic region, n (%)			0.00
South	65 701 (35.3)	65 701 (35.3)	
North Central	55 756 (29.9)	55 756 (29.9)	
Northeast	41 929 (22.5)	41 929 (22.5)	
West	22 916 (12.3)	22 916 (12.3)	
CCI score, mean (SD)	1.07 (1.5)*	0.98 (1.5)	-0.06
CCI score, n (%)		*	0.08
0	93 639 (50.2)	99 666 (53.5)	
1	39 806 (21.4)	39 933 (21.4)	
2	26 506 (14.2)	23 492 (12.6)	
≥3	26 423 (14.2)	23 283 (12.5)	
Any evaluated CCI condition^c^	92 735 (49.8)*	86 708 (46.5)	-0.06
Chronic pulmonary disease	32 204 (17.3)*	26 166 (14.0)	-0.09
Diabetes without chronic complication	31 235 (16.8)*	28 799 (15.5)	-0.04
Any malignancy including leukemia and lymphoma	18 158 (9.7)*	14 057 (7.5)	-0.08
Peripheral vascular disease	12 856 (6.9)	12 861 (6.9)	0.00
Cerebrovascular disease	12 080 (6.5)*	13 033 (7.0)	0.02
Any prespecified comorbidity	70 160 (37.6)*	60 710 (32.6)	-0.11
Sleep-related conditions	31 335 (16.8)*	26 130 (14.0)	-0.08
Obesity	27 717 (14.9)*	24 720 (13.3)	-0.05
Depression	21 283 (11.4)*	15 710 (8.4)	0.00
Anxiety	19 986 (10.7)*	17 072 (9.2)	-0.01

*P<0.0001.

Abbreviations: CCAE, Commercial Claims and Encounters Database; CCI, Charlson Comorbidity Index; MDCR, Medicare Supplemental and Coordination of Benefits Database; Std Diff, standard difference.

^a^ Data for each comorbidity were derived from International Statistical Classification of Diseases and Related Health Problems codes. Each represents a CCI comorbidity or other pre-specified comorbidity of interest.

^b^ Shown for health plan types with ≥10% of patients.

^c^ CCI comorbidities with a prevalence ≥5% in the OA pain cohort.

The mean CCI score was significantly higher in the MTS OA pain cohort compared with non-MTS OA pain (1.07 vs 0.98; *P*<0.0001), and the distribution of scores, which was significantly different between the cohorts (*P*<0.0001), indicated a trend toward a greater comorbidity burden in the MTS OA pain cohort (Table 1). Significantly higher proportions of patients with MTS OA pain were observed for any evaluated CCI-related conditions compared with patients with non-MTS OA pain (49.8% vs 46.5%), including chronic pulmonary disease, diabetes without chronic complications, and any malignancies including leukemia and lymphoma (all *P*<0.0001). However, a significantly lower proportion of patients with MTS OA pain had cerebrovascular disease than patients with non-MTS OA pain (6.5% vs 7.0%; *P*<0.0001). The MTS OA pain cohort had a significantly higher prevalence of prespecified comorbidities overall compared with the non-MTS OA pain cohort (37.6% vs 32.6%; *P*<0.0001), including sleep-related conditions, obesity, depression, and anxiety (all *P*<0.0001).

### Treatment Patterns

A significantly greater proportion of patients with MTS OA pain was prescribed pain medications at baseline compared with the non-MTS OA pain cohort (77.0% vs 68.3%; *P*<0.0001). Furthermore, all evaluated medications (**Supplemental Table 3**) were prescribed to significantly higher proportions of patients with MTS OA pain vs non-MTS OA pain at baseline. Of these medications, the most prescribed in the MTS vs non-MTS OA pain cohorts were nontramadol opioids (41.2% vs 28.6%), NSAIDs/COX-2 inhibitors (39.5% vs 32.3%), antidepressants (28.3% vs 21.7%), benzodiazepines (23.3% vs 17.9%), and anticonvulsants (21.9% vs 16.8) (all *P*<0.0001; Table 2). Similarly, significant differences were observed across all classes of prescribed pain medications at 12 and 24 months, with higher proportions of medication usage in the MTS OA pain cohort (Table 2). Prescription pain medications were 10%-20% higher in the MTS OA pain cohort compared with non-MTS OA pain cohort overall at the 12-month and 24-month follow-up periods relative to baseline. The same 5 classes of medications were most prescribed at both follow-up periods for patients with MTS OA pain, which included IA corticosteroids/HA that showed a 4.1% greater usage at baseline, 13.9% at 12 months, and 14.3% at 24 months compared with patients with non-MTS pain.

**Table 2.** Patients (%) with Pain Medication Prescriptions at Baseline and 12- and 24-Month Follow-up in the Propensity Score–Matched Cohorts^a^

**Pain Medication**	**Baseline^b^**			**12-Month Follow-up**			**24-Month Follow-up**		
	**MTS**	**Non-MTS**	**SD**	**MTS**	**Non-MTS**	**Std Diff**	**MTS**	**Non-MTS**	**Std Diff**
Any	143 492 (77.0)*	127 212 (68.3)	-0.20	166 943 (89.6)*	141 848 (76.1)	-0.36	175 246 (94.0)*	158 505 (85.1)	-0.30
Analgesics/antipyretics	14 910 (8.0)*	11 646 (6.3)	-0.07	18 522 (9.9)*	12 572 (6.8)	-0.12	27 378 (14.7)*	19 811 (10.6)	-0.12
NSAIDs/COX-2 inhibitors	73 608 (39.5)*	60 158 (32.3)	-0.15	98 725 (53.0)*	74 930 (40.2)	-0.26	117 340 (63.0)*	94 956 (51.0)	-0.24
IA corticosteroids/HA	23 952 (12.9)*	16 299 (8.8)	-0.13	55 948 (30.0)*	30 719 (16.5)	-0.32	68 327 (36.7)*	41 724 (22.4)	-0.32
Tramadol	25 316 (13.6)*	16 098 (8.6)	-0.16	39 154 (21.0)*	18 743 (10.1)	-0.31	51 085 (27.4)*	28 310 (15.2)	-0.30
Non-tramadol opioids	76 721 (41.2)*	53 226 (28.6)	-0.27	106 343 (57.1)*	54 634 (29.3)	-0.58	127 690 (68.5)*	79 160 (42.5)	-0.54
Antidepressants	52 659 (28.3)*	40 370 (21.7)	-0.15	55 425 (29.7)*	43 103 (23.1)	-0.15	63 818 (34.2)*	50 879 (27.3)	-0.15
Muscle relaxants	28 697 (15.4)*	21 477 (11.5)	-0.11	32 414 (17.4)*	22 551 (12.1)	-0.15	46 237 (24.8)*	34 449 (18.5)	-0.15
Benzodiazepines	43 376 (23.3)*	33 415 (17.9)	-0.13	51 008 (27.4)*	36 085 (19.4)	-0.19	67 068 (36.0)*	51 337 (27.6)	-0.18
Anxiolytics/sedatives/hypnotics	21 787 (11.7)*	15 540 (8.3)	-0.11	23 122 (12.4)*	15 287 (8.2)	-0.14	30 177 (16.2)*	20 709 (11.1)	-0.15
Anticonvulsants	40 745 (21.9)*	31 352 (16.8)	-0.13	53 300 (28.6)*	36 842 (19.8)	-0.21	72 412 (38.9)*	54 643 (29.3)	-0.20

*P<0.0001.

Abbreviations: COX, cyclooxygenase; HA, hyaluronic acid; IA intra-articular; NSAIDs, nonsteroidal anti-inflammatory drugs; OA, osteoarthritis; SD, standard difference.

^a^n=186 374 per cohort. Values are presented as number (%) of patients.

^b^ Baseline is the 12 months prior to index. Any prescriptions during the 12- and 24-month follow-up were included in the 12- and 24-month data, respectively.

**Figure 1.**
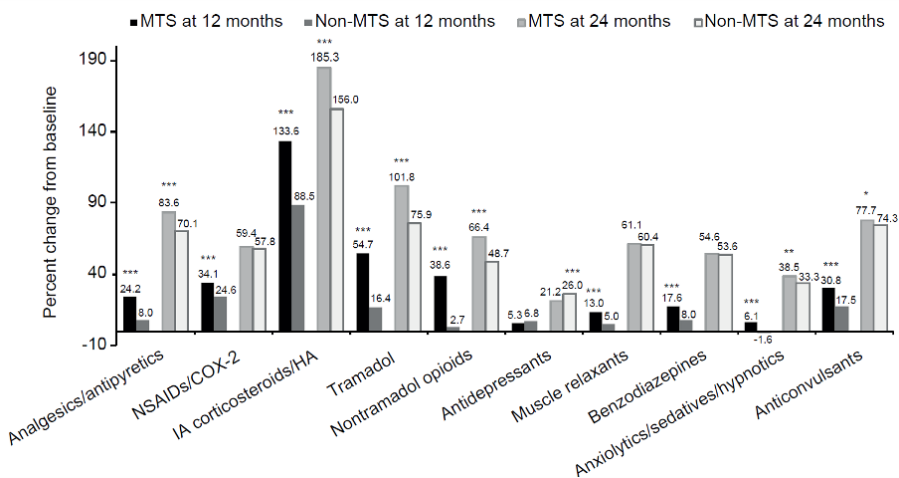
Change in Patients Prescribed Pain Medications at 12 and 24 Months in Propensity Score–Matched Cohorts Percent change from baseline in patients prescribed pain medications at 12 and 24 months in the propensity score-matched cohorts (n=186 374 per cohort). *P<0.05; **P<0.01; ***P<0.0001 by chi-square test between cohorts at 12 and 24 months. Abbreviations: COX, cyclooxygenase; HA, hyaluronic acid; IA, intra-articular; MTS, moderate to severe; NSAIDs, nonsteroidal anti-inflammatory drugs; OA, osteoarthritis.

Examination of changes from baseline to the 12-month follow-up showed that the MTS OA pain cohort had significantly greater increases in use of all classes of prescription pain medications compared with non-MTS OA pain cohort (*P*<0.0001), except for antidepressants (Figure 1). Medications with the greatest increase in use at the 12-month follow-up were IA corticosteroid/HA injections (133.6% MTS vs 88.5% non-MTS), tramadol (54.7% vs 16.4%), nontramadol opioids (38.6% vs 2.7%), NSAIDs/COX-2 inhibitors (34.1% vs 24.6%), anticonvulsants (30.8% vs 17.5%), and analgesics/antipyretics (24.2% vs 8%) (all *P*<0.0001; Figure 1).

Examination of changes from baseline to the 24-month follow-up revealed generally similar patterns for patients with MTS OA pain as that at 12 months, although not all differences between cohorts were statistically significant. Significantly higher changes in the MTS pain cohort were observed, in decreasing order, for IA corticosteroid/HA injections, tramadol, analgesics/antipyretics, anticonvulsants, nontramadol opioids, and anxiolytics/sedatives/hypnotics (all *P*<0.05). In contrast, the MTS OA pain cohort had significantly lower antidepressant use (21.2%) at 24 months compared with the non-MTS cohort (26.0%) (*P*<0.0001).

### Medication Adherence

**Figure 2.**
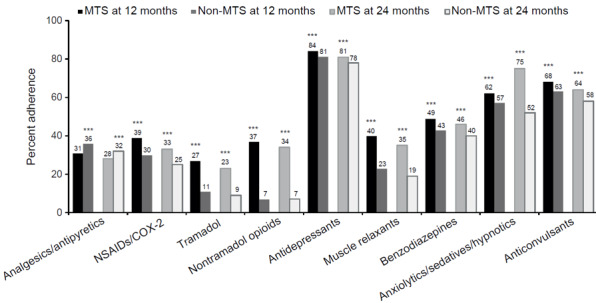
PDC of Prescribed Oral Pain Medications at 12 and 24 Months in Propensity Score–Matched OA Cohorts PDC of prescribed oral pain medications at 12 and 24 months in the propensity-matched OA cohorts initiating a medication within 30 days post-index (n=186 374 per cohort). ***P<0.0001. Abbreviations: COX, cyclooxygenase; MTS, moderate to severe; NSAIDs, nonsteroidal anti-inflammatory drugs; OA, osteoarthritis; PDC, proportion of days covered.

Medication adherence, as measured by PDC, was highest at the 12- and 24-month follow-ups for antidepressants in both OA pain cohorts (78%-84%), followed by anticonvulsants (58%-68%) and anxiolytics/sedatives/hypnotics (33%-62%) (Figure 2). At both 12 and 24 months, patients with MTS OA pain had significantly higher PDC compared with patients with non-MTS OA pain for all pain medication types assessed (all *P*<0.0001), except for analgesics/antipyretics, in which patients with MTS OA pain had significantly lower PDC compared with patients with non-MTS OA pain (31% vs 36% at 12 months; 28% vs 32% at 24 months; *P*<0.0001). The largest differences in PDC between the 2 cohorts were observed for non-tramadol opioids: 37% in the MTS cohort vs 7% in the non-MTS cohort at 12 months and 34% vs 7%, respectively, at 24 months (both *P*<0.001).

### Resource Utilization

Significantly greater HCRU was observed for patients with MTS OA pain compared with non-MTS for all resource categories examined, including inpatient hospitalizations, outpatient visits, ER visits, and rehabilitation and physical therapy (PT) visits for baseline and 12- and 24-month follow-up periods (all *P*<0.0001; Table 3). In patients with MTS OA pain, outpatient visits increased from mean (SD) of 22.8 (22.4) at baseline to 32.9 (26.6) and 60.7 (47.8) at the 12- and 24-month follow-ups, respectively. For patients with MTS OA pain, rehabilitation and PT visits increased from 1.5 (5.3) at baseline to 3.8 (8.6) and 6.2 (12.8) at 12 and 24 months, respectively; similarly, ER visits increased from 0.5 (1.2) visits at baseline to 0.6 (1.4) and 1.1 (2.4) at 12 and 24 months, respectively (all *P*<0.0001). Length of hospital stay was significantly longer at 12 and 24 months for the MTS cohort compared with the non-MTS cohort, as were hospital readmissions (both *P*<0.0001). Hospital readmissions increased in the MTS cohort from 1236 patients (0.7%) at baseline to 2509 (1.4%) and 4262 (2.3%) patients at the 12- and 24-month follow-ups, respectively. Increases were also observed for the non-MTS OA pain cohort, although to a lesser extent (Table 3). The number of filled prescriptions for each pain medication type was significantly greater in patients with MTS OA pain compared with non-MTS OA pain across the baseline and both follow-up periods (all *P*<0.0001). In patients with MTS OA pain, the mean (SD) of filled prescriptions increased from 28.7 (26.8) at baseline to 32.2 (28.2) at 12 months and to 63.9 (55.6) at 24 months. A similar pattern of increase over time was observed for patients with non-MTS OA pain but to a lesser extent than patients with MTS OA pain (Table 3).

**Table 3.** Healthcare Resource Utilization at Baseline and 12- and 24-Month Follow-up in Propensity Score–Matched Cohorts

**Resource Category**	**Baseline**			**12-Month Follow-up**			**24-Month Follow-up**		
	**MTS**	**Non-MTS**	**Std Diff**	**MTS**	**Non-MTS**	**Std Diff**	**MTS**	**Non-MTS**	**Std Diff**
Inpatient hospitalizations	0.1±0.4**	0.1**±**0.4	-0.03	0.3±0.6**	0.1**±**0.5	-0.32	0.5±0.9**	0.3**±**0.7	-0.34
Readmissions, n (%)	1236 (0.7)*	1058 (0.6)	-0.01	2509 (1.4)**	1272 (0.7)	-0.07	262 (2.3)**	2335 (1.3)	-0.08
Hospitalization length, days	0.5**±**2.0	0.5**±**2.2	0.00	1.0±2.5**	0.5**±**2.3	-0.21	1.5±2.9**	0.9**±**2.8	-0.22
Outpatient visits	22.8±22.4**	19.6 21.1	-0.15	32.9±26.6**	25.3**±**24.2	-0.30	60.7±47.8**	47.6**±**44.4	-0.29
ER visits	0.5±1.2**	0.43**±**1.1	-0.03	0.6±1.4**	0.5**±**1.3	-0.05	1.1±2.4**	1.0**±**2.2	-0.05
Rehabilitation and PT visits	1.5±5.3**	1.1**±**4.8	-0.06	3.8±8.6**	2.2**±**6.5	-0.21	6.2±12.8**	3.45**±**9.6	-0.24
Filled prescriptions	28.7±26.8**	22.3**±**21.4	-0.26	32.2±28.2**	24.3**±**22.6	-0.31	63.±55.6**	49.0**±**45.1	-0.29

Baseline is the 12 months prior to index. Percent (%) refers to users, ie, patients with >1 of each of the characteristics (n>1); n=186374 per cohort. Values are presented as mean ± SD unless otherwise indicated.

*P<0.01; **P<0.0001.

Abbreviations: ER, emergency room; OA, osteoarthritis; PT, physical therapy; Std Diff, standard difference.

### Direct Medical Costs

**Figure 3.**
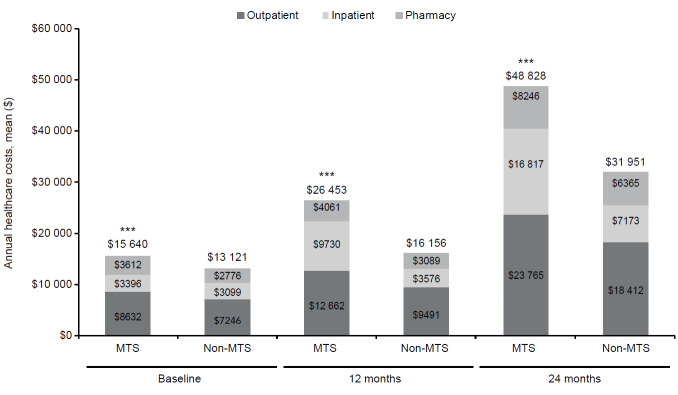
Direct Medical Costs at Baseline, 12, and 24 Months in Propensity Score–Matched OA Pain Cohorts Direct medical costs at baseline, 12, and 24 months in the propensity score–matched OA pain cohorts (n=186 374 per cohort) The number on top of each bar represents the total healthcare costs, which is a sum of inpatient, outpatient, and pharmacy costs. ***P<0.0001. Abbreviations: MTS, moderate to severe; OA, osteoarthritis.

The patients with MTS OA pain had significantly higher mean total healthcare costs overall and for the individual cost components of outpatient, inpatient, and pharmacy categories compared with patients with non-MTS OA at baseline and the 12- and 24-month follow-ups (all *P*<0.0001) (Figure 3). Total mean (SD) health care costs were $15 640 ($35 878) vs $13 121 ($37 094) at baseline, $26 453 ($47 287) vs $16 156 ($40 128) at 12 months, and $48 828 ($83 190) vs $31 951 ($76 128) at 24 months, respectively, for MTS vs non-MTS OA pain cohorts (all *P*<0.0001). The total costs in the MTS OA pain cohort were higher than non-MTS OA pain by 19% at baseline, 64% at 12 months, and 53% at 24 months.

Across all time periods and cohorts, total costs were primarily driven by outpatient costs. At baseline, outpatient costs accounted for the majority of costs (55%) for both OA pain cohorts, as shown in Figure 3. The second largest contributor of total costs were inpatient costs across all time periods and cohorts, with the exception of baseline costs for patients with MTS OA pain in which mean pharmacy costs were $3612 ($9914) and inpatient costs were $3396 ($20 568). This contrasted with patients with non-MTS OA, in which mean inpatient costs were $3099 ($22 216) and pharmacy costs were $2776 ($8252).

For both the 12- and 24-month follow-up periods, outpatient costs continued to be the main driver of total healthcare costs in both cohorts, although the proportion relative to total costs decreased over time in patients with MTS OA pain but increased in the non-MTS cohort. Furthermore, inpatient costs, relative to total costs, increased over time in patients with MTS OA pain, whereas pharmacy costs decreased (Figure 3). There was little change in the proportional contribution of inpatient and pharmacy costs to total costs over time in the non-MTS OA pain cohort.

## DISCUSSION

This study contributes important longitudinal information on the clinical and economic burden of OA, by pain severity level, spanning 24 months of follow-up post-OA diagnosis. It relies on a unique algorithm that allows healthcare researchers to identify patients with more severe OA pain while utilizing administrative claims data to track resource utilization and costs. Mean age in the PSM cohorts was 63 years, with approximately two-thirds of patients of working age and enrolled in commercial plans. Patients with MTS OA pain in our study had a significantly greater comorbidity burden compared with patients with non-MTS pain, including sleep disturbance, insomnia, depression, and anxiety, not unlike what has been reported in other OA studies using cross-sectional survey data in patients with increasing pain severity or pain interference with activities (PIA).^17,46,54^ None of those studies, however, evaluated longitudinal changes in comorbidities over time.

Prescription pain medication use for OA showed a similar pattern to comorbidities, with significantly increased use of OA medications in patients with MTS OA pain relative to patients with non-MTS OA pain. Although the high prescribing of OA pain medications at baseline may be related to treatment of conditions other than OA, it is also possible that the patient was being treated for OA in the absence of an OA diagnosis during the baseline period. In patients with MTS OA pain, baseline prescribing was highest for nontramadol opioids (41.2%) followed by NSAIDs/ COX-2 inhibitors (39.5%). The use of NSAIDs is in line with current treatment guidelines for OA. The high use of non- tramadol opioids contrasts with the conditional recommendation to start tramadol if an opioid is necessary, and only choose non-tramadol opioids when all other treatment options have failed.^20^ However, the recent guidelines postdate the years of data analyzed in the current study (2013-2018), and the treatment patterns we observed may reflect recommendations from earlier guidelines. Significantly greater prescription pain medication use for OA was reported by Schepman et al^54^ in MTS pain respondents compared with mild pain (41% vs 17%; *P*<0.0001), which is approximately half the proportions in the current study, but may not be unexpected as their study was based on patient self-report rather than administrative claims. In their study, non-tramadol opioids and NSAIDs were the most prevalent classes prescribed among MTS OA pain respondents (18% each cohort). Nalamachu et al^18^ examined physician-reported prescription painmedications for a cohort of patients with OA stratified by patient-reported OA pain severity and reported proportions of current medications for OA (mild [67.3%] and moderate [80.8%] vs severe [77.7%]) relative to the 77% in patients with MTS OA pain and 68.3% in non-MTS OA pain in our study. The most commonly prescribed pain medications reported by Nalamachu et al^18^ in the moderate or severe pain cohorts were NSAIDs (51%-62.3%) followed by opioids (26.5%-32.5%). These results are similar to ours despite using very different methodologies and may provide supporting evidence for the patient identification algorithm employed in our study.

A unique aspect of this study is that it provides insights into the current real-world prescribing of pain medications for MTS OA by following patients for 2 years. Significantly greater pain medication use was observed for patients with MTS OA pain compared with non-MTS at 12 months for all pain medication types, except antidepressants. This trend was also observed at 24 months in which all medication types except NSAID/COX-2 inhibitors, benzodiazepines, and muscle relaxants were significantly greater in the MTS OA pain cohort. Although 2 studies reported on the change in OA pain medications over time, neither stratified patients by OA pain severity.^26,27^ One of these studies, using data from the Osteoarthritis Initiative, reported lower medication use than observed in the current study, and no significant changes over the 36-month time period, with the proportion of patients using acetaminophen or NSAIDs remaining at similar levels (acetaminophen, 12.5%-14.4%; NSAIDs, 8.1%-9.3%).^26^ The second study, using 2-year data from the Medical Expenditure Panel Survey (MEPS), examined the relationship between opioid use patterns defined as persistent, intermittent, and no use, and PIA.^27^ The authors found that patients with persistent opioid use had poorer PIA, suggesting the potential for worse outcomes with chronic opioid use.

Our study showed significantly greater adherence, measured by PDC, to prescription OA pain medications in patients with MTS OA pain vs non-MTS for all medication types. Although PDC at the 12- and 24-month follow-ups was highest for antidepressants, anticonvulsants, and anxiolytics/sedatives/hypnotics in both OA cohorts, a PDC ≥80%, defined as the threshold for being considered adherent, was only observed with antidepressants across both OA pain severity cohorts. Using PDC as a proxy for adherence, this is not surprising given antidepressants are also used for symptoms beyond OA pain where high adherence is often observed. Pain medications, on the other hand, are often used as needed, and it may be possible that the lower PDCs observed in this study represent numerically lower yet appropriate rates of adherence. PDC is a useful measure in that, regardless of as-needed or scheduled use, it informs the level of utilization of a particular medication or class of medications. No studies of medication adherence were identified in OA, although several studies reported suboptimal treatment adherence in patients with other rheumatic diseases.^36–38^ Adherence (PDC ≥80%) was found to range from 18%-46% for 5 different biologics at 12 months post-initiation in patients with psoriatic arthritis.^38^

HCRU was significantly greater for patients with MTS OA pain compared with non-MTS for inpatient hospitalizations, readmissions, outpatient, ER, and rehabilitation and PT visits. Somewhat similar findings were observed by Schepman et al,^54^ in which significantly higher proportions of outpatient visits, ER visits, and hospitalizations were reported in the past 6 months for survey respondents with MTS OA pain relative to mild OA pain. Nalamachu et al^46^ showed that physician- and patient-reported healthcare visits in the past 3 months and hospitalizations in the past 12 months significantly increased at higher levels of self-reported OA pain severity. Zhao et al^17^ reported significantly higher proportions of patients with hospitalizations, ER visits, and specialist visits among adults with OA who reported moderate/severe PIA compared with no/mild PIA, independent of opioid use (all *P*<0.01). Lastly, Wei et al^45^ reported significantly greater frequency of OA-related resource use including ER visits, outpatient office visits, and hospitalizations with increasing pain episodes.

The economic burden of OA, especially in patients with MTS OA pain, is substantial. Our findings showed significantly higher costs in the MTS OA pain cohort at baseline and at both 12- and 24-month follow-ups compared with the non-MTS cohort, with increasing costs for each of the cohorts over time. In 2 published studies, higher healthcare costs were significantly associated with increasing PIA^17^ and pain episodes^45^ in a manner similar to our findings, using different patient identification, pain definition, and costing algorithms. Using MEPS data from 2011/2013/2015, Zhao et al^17^ reported mean (standard error) total costs of $8730 ($642) in a moderate/severe PIA OA cohort with no opioid use and $18 613 ($1116) for moderate/severe PIA OA adults with opioid use. The opioid cohort total expenditures are similar to the $15 640 ($35 878) reported in the MTS OA pain cohort at baseline in this study, which is not surprising given that one of the patient identification criteria included prescription opioid use. These studies were cross-sectional and conducted at a single point in time, precluding longitudinal comparison with our findings.

In the current study, 63% of the patients with OA were categorized as having MTS OA pain. This proportion is similar to the 65% reported by Schepman et al^54^ and 55% reported by Nalamachu et al^18,46^ that assessed OA pain severity using the 2019 National Health and Wellness Survey and 2017 US Adelphi Disease Specific Programme data sources, respectively. Our definition of MTS OA pain was derived using a claims-based algorithm, which differed from the other studies that stratified OA pain severity using patient-reported measures. Claims data do not capture information on OA pain or pain severity and rely on the use of diagnostic codes for patient identification and may be subject to coding errors. Although the strength of the algorithm is linked to its application to administrative claims data, it is a less precise method than capturing OA pain severity using self-reported methodologies that are the mainstay in patient and physician surveys. Additional validation of the algorithm employed in this study to ensure identification of the appropriate patient population should be conducted.

Several study limitations should be considered. Although providing a wealth of clinical and economic information, claims data are limited in that medication use cannot be attributed to a specific disease, so pain medications prescribed within 30 days of index may not have been for OA. Claims data also do not capture information on patients change of healthcare insurance or if they have multiple insurance plans at the same time. Some prescription pain medications commonly used to treat OA also treat a number of other illnesses including depression, anxiety, and epilepsy. Given that patients with OA, especially those with more advanced disease, have a high comorbidity burden, these medications could be prescribed for other underlying conditions. Despite the strengths of our approach (ie, using a validated claims-based algorithm and real-world data from a large healthcare database), there is an overlap of certain variables included in the definition of the cohorts and the outcomes analyzed. However, as the goal was to describe overall utilization, this study used a narrow timeline to define the variables included in the definition of the cohorts the length in time for the outcomes. Claims data also do not capture information on nonpharmacologic therapies and over-the-counter medications used to treat OA pain, nor are indirect costs, such as those related to lost productivity at work, absenteeism, and caregiver burden, captured. Therefore, the total costs reported in our population are likely underestimated. Although the sample size in our study was robust, patients were required to be continuously enrolled for 3 years in a health plan. Such patients may differ from other plan members; hence, generalizability beyond this commercially insured population may be limited. These results are not representative of all patients with OA. This study included only commercial insured patients with private or Medicare plus supplemental insurance that were seeking medical care. Generalizability may have been impacted when we applied PSM, as very healthy and very severe patients would have been excluded from the final cohorts. Claims data, however, are a rich source of information and, when used alongside other methodologies, expand our understanding of the clinical and economic impact of patients with MTS OA pain.

## CONCLUSIONS

The clinical and economic burden observed in this MTS OA population suggests a high unmet need that may require development of novel therapies or strategies to treat OA pain and improved healthcare strategies to manage disease progression. Patients with OA do not represent a homogeneous population, and focusing on population subtypes such as pain severity will allow a more targeted and efficient approach to patient management. Further research is warranted to examine the factors contributing to the substantial burden in this more severe OA pain population.

### Disclosures

The study was sponsored by Pfizer and Eli Lilly and Company. The findings of this manuscript have been presented at The International Osteoporosis Foundation (IOF) and the European Society for Clinical and Economic Aspects of Osteoporosis and Osteoarthritis (ESCEO) 2020 Virtual World Congress (WCO-IOF-ESCEO); August 20-22, 2020, and at PAINWeek 2020 Virtual Meeting; September 11-13, 2020. The following authors are employees of Pfizer with stock and/or stock options: PS, ST, CB, DM, and BE. RLR is an employee and stockholder of Eli Lilly and Company. RNH received consulting fees associated with this study from Pfizer and Eli Lilly and Company. The authors report no conflicts of interest in this work.

## Supplementary Material

Supplementary Online Material
