# Supplementary material for: A Retrospective Claims-Based Study Evaluating Clinical and Economic Burden Among Patients With Moderate to Severe Osteoarthritis Pain in the United States
Source: J Health Econ Outcomes Res. 2022 Mar 1;9(1):58–67. doi: 10.36469/jheor.2022.31895 (PMC8888122; doi:10.36469/jheor.2022.31895)
Supplement: Supplementary Online Material [file jheor_2022_9_1_31895_83188.pdf]

Journal of Health Economics and Outcomes Research

Online Supplementary Material

A Retrospective Claims-Based Study Evaluating Clinical and Economic Burden Among Patients With Moderate to Severe Osteoarthritis Pain in the United States. *JHEOR*. **2022;9(1):58-67.** doi:10.36469/jheor.20XX

Figure S1: Patient Disposition

Table S1: Diagnosis and Procedural Codes Used to Define Moderate to Severe Osteoarthritis Pain Cohort

Table S2: Definition of the Outcomes

Table S3: Pain Medications by Class

This supplementary material has been provided by the authors to give readers additional information about their work.

This is an open-access article distributed under the terms of the Creative Commons Attribution 4.0 International License (CCBY-4.0). View this license's legal deed at http://creativecommons.org/licenses/by/4.0 and legal code at http://creativecommons.org/licenses/by/4.0/legalcode for more information.

Figure S1. Patient Disposition

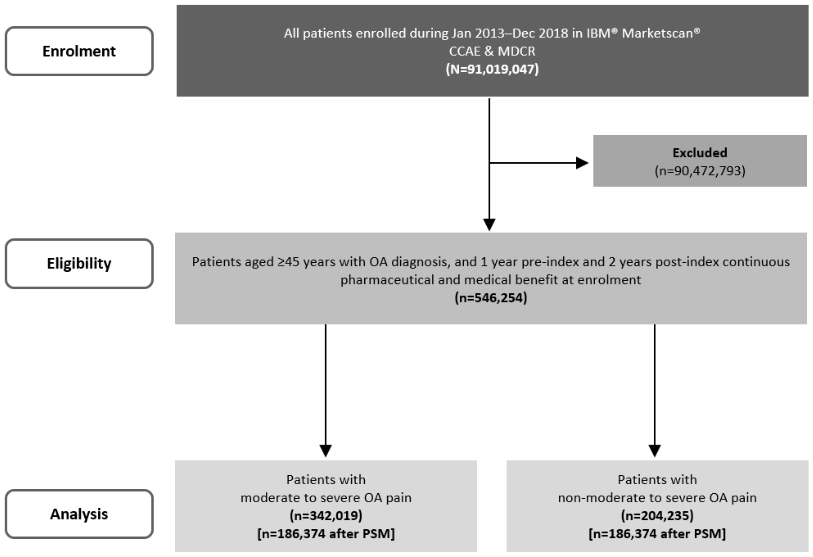

Table S1. Diagnosis and Procedural Codes Used to Define Moderate to Severe Osteoarthritis Pain Cohort

ICD-10 Codes	Diagnoses
M150	Primary generalized (osteo)arthritis
M151	Heberden's nodes (with arthropathy)
M152	Bouchard's nodes (with arthropathy)
M153	Secondary multiple arthritis
M158	Other polyosteoarthritis
M159	Polyosteoarthritis, unspecified
M1610	Unilateral primary osteoarthritis, unspecified hip
M1611	Unilateral primary osteoarthritis, right hip
M1612	Unilateral primary osteoarthritis, left hip
M1630	Unilateral osteoarthritis resulting from hip dysplasia, unspecified hip
M1631	Unilateral osteoarthritis resulting from hip dysplasia, right hip
M1632	Unilateral osteoarthritis resulting from hip dysplasia, left hip
M164	Bilateral post-traumatic osteoarthritis of hip
M1650	Unilateral post-traumatic osteoarthritis, unspecified hip
M1651	Unilateral post-traumatic osteoarthritis, right hip
M1652	Unilateral post-traumatic osteoarthritis, left hip
M166	Other bilateral secondary osteoarthritis of hip
M167	Other unilateral secondary osteoarthritis of hip
M169	Osteoarthritis of hip, unspecified
M170	Bilateral primary osteoarthritis of knee
M1710	Unilateral primary osteoarthritis, unspecified knee

M1711	Unilateral primary osteoarthritis, right knee
M1712	Unilateral primary osteoarthritis, left knee
M172	Bilateral post-traumatic osteoarthritis of knee
M1730	Unilateral post-traumatic osteoarthritis, unspecified knee
M1731	Unilateral post-traumatic osteoarthritis, right knee
M1732	Unilateral post-traumatic osteoarthritis, left knee
M174	Other bilateral secondary osteoarthritis of knee
M175	Other unilateral secondary osteoarthritis of knee
M179	Osteoarthritis of knee, unspecified
M1990	Unspecified osteoarthritis, unspecified site
M1991	Primary osteoarthritis, unspecified site
M1993	Secondary osteoarthritis, unspecified site
M25551	Pain in right hip
M25552	Pain in left hip
M25559	Pain in unspecified hip
M25561	Pain in right knee
M25562	Pain in left knee
M25569	Pain in unspecified knee

**ICD-9 Codes**	**Diagnoses**
71500	Osteoarthrosis generalized involving unspecified site
71509	Osteoarthrosis generalized involving multiple sites
71510	Osteoarthrosis localized primary involving unspecified site
71515	Osteoarthrosis localized primary involving pelvic region and thigh
71516	Osteoarthrosis localized primary involving lower leg
71518	Osteoarthrosis localized primary involving other specified sites
71520	Osteoarthrosis localized secondary involving unspecified site
71525	Osteoarthrosis localized secondary involving pelvic region and thigh
71526	Osteoarthrosis localized secondary involving lower leg
71528	Osteoarthrosis localized secondary involving other specified sites
71530	Osteoarthrosis localized not specified whether primary or secondary involving unspecified site
71535	Osteoarthrosis localized not specified whether primary or secondary involving pelvic region and thigh
71536	Osteoarthrosis localized not specified whether primary or secondary involving lower leg
71538	Osteoarthrosis localized not specified whether primary or secondary involving other specified sites
71580	Osteoarthrosis involving or with more than one site but not specified as generalized and involving unspecified site
71589	Osteoarthrosis involving or with multiple sites but not specified as generalized
71590	Osteoarthrosis unspecified whether generalized or localized involving unspecified site
71595	Osteoarthrosis unspecified whether generalized or localized involving pelvic region and thigh
71596	Osteoarthrosis unspecified whether generalized or localized involving lower leg
71598	Osteoarthrosis unspecified whether generalized or localized involving other specified sites

**CPT4 Code**	**Procedure Type**
27054	arthrotomy with synovectomy, hip joint
27125	hemiarthroplasty, hip, partial (eg, femoral stem prosthesis, bipolar arthroplasty)
27130	arthroplasty, acetabular and proximal femoral prosthetic replacement (total hip arthroplasty), with or without autograft or allograft
27132	conversion of previous hip surgery to total hip arthroplasty, with or without autograft or allograft

27134	revision of total hip arthroplasty; both components, with or without autograft or allograft
27138	revision of total hip arthroplasty; femoral component only, with or without allograft
27284	arthrodesis, hip joint (including obtaining graft)
27286	arthrodesis, hip joint (including obtaining graft); with subtrochanteric osteotomy
27332	arthrotomy, with excision of semilunar cartilage (meniscectomy) knee; medial or lateral
27333	arthrotomy, with excision of semilunar cartilage (meniscectomy) knee; medial and lateral
27347	excision of lesion of meniscus or capsule (eg, cyst, ganglion), knee
27403	arthrotomy with meniscus repair, knee
27440	arthroplasty, knee, tibial plateau
27441	arthroplasty, knee, tibial plateau; with debridement and partial synovectomy
27442	arthroplasty, femoral condyles or tibial plateau(s), knee
27443	arthroplasty, femoral condyles or tibial plateau(s), knee; with debridement and partial synovectomy
27445	arthroplasty, knee, hinge prosthesis (eg, Walldius type)
27446	arthroplasty, knee, condyle and plateau; medial or lateral compartment
27447	arthroplasty, knee, condyle and plateau; medial and lateral compartments with or without patella resurfacing (total knee arthroplasty)
27486	revision of total knee arthroplasty, with or without allograft; 1 component
29860	arthroscopy, hip, diagnostic with or without synovial biopsy (separate procedure)
29862	arthroscopy, hip, surgical; with debridement/shaving of articular cartilage (chondroplasty), abrasion arthroplasty, and/or resection of labrum
29863	arthroscopy, hip, surgical; with synovectomy
29868	arthroscopy, knee, surgical; meniscal transplantation (includes arthrotomy for meniscal insertion), medial or lateral
29870	arthroscopy, knee, diagnostic, with or without synovial biopsy (separate procedure)
29877	arthroscopy, knee, surgical; debridement/shaving of articular cartilage (chondroplasty)
29879	arthroscopy, knee, surgical; abrasion arthroplasty (includes chondroplasty where necessary) or multiple drilling or microfracture
29880	arthroscopy, knee, surgical; with meniscectomy (medial and lateral, including any meniscal shaving) including debridement/shaving of articular cartilage (chondroplasty), same or separate compartment(s), when performed
29881	arthroscopy, knee, surgical; with meniscectomy (medial or lateral, including any meniscal shaving) including debridement/shaving of articular cartilage (chondroplasty), same or separate compartment(s), when performed
29882	arthroscopy, knee, surgical; with meniscus repair (medial or lateral)
29883	arthroscopy, knee, surgical; with meniscus repair (medial and lateral)
97810	acupuncture, 1 or more needles; without electrical stimulation, initial 15 minutes of personal one-on-one contact with the patient
97811	acupuncture, 1 or more needles; without electrical stimulation, each additional 15 minutes of personal one-on-one contact with the patient, with re-insertion of needle(s) (list separately in addition to code for primary procedure)
97813	acupuncture, 1 or more needles; with electrical stimulation, initial 15 minutes of personal one-on-one contact with the patient
97814	acupuncture, 1 or more needles; with electrical stimulation, each additional 15 minutes of personal one-on-one contact with the patient, with re-insertion of needle(s) (list separately in addition to code for primary procedure)

Comorbidities of Interest	ICD-9 and ICD-10 Codes
Sleep-related conditions	Any of the following: organic sleep disorders (327.0x-327.1x), specific disorders of sleep of nonorganic origin (307.4x); sleep disturbances (780.5x); organic sleep disorders (327.x); cataplexy and narcolepsy (347.x); and lack of adequate sleep (V69.4).
Obesity	Any of the following ICD9: 278.0x-278.03x, 7831, V553, V4586<br><br>Any of the following ICD10: E65, E66, E660, E6601, E6609, E661, E662, E663, E668, E669, E670
Anxiety	Any of the following ICD9: 300.0x, 300.1x, 300.2x, 300.3-300.3-300.7, 300.8x<br><br>Any of the following ICD10: F40, F400, F4000, F4001, F4002, F401, F4010, F4011, F402, F4021, F40210, F40218, F4022, F40220, F40228, F4023, F40230, F40231, F40232, F40233, F4024, F40240, F40241, F40242, F40243, F40248, F4029, F40290, F40291, F40298, F408, F409, F41, F410, F411, F413,<br><br>F418, F419, F42, F422, F423, F424, F428, F429, F44.x, F45.x
Depression	Any of the following ICD9: major depressive disorder (296.2x, 296.3x) or other depressive symptoms (311.xx)<br><br>Any of the following ICD10: F32, F320, F321, F322, F323, F324, F325, F328, F3281, F3289, F329, F33, F330, F331, F332, F333, F334, F3340, F334, F3342, F338, F339
OCD	Any of the following ICD9: 300.3<br><br>Any of the following ICD10: F42, F422, F423, F424, F428, F429, R4681
PTSD	Any of the following ICD9: 309.81<br><br>Any of the following ICD10: F431, F4310, F4311, F4312

Table S2. Pain Medications by Class

Outcomes	Definition
Inpatient hospitalizations	Number of admissions to inpatient service of a hospital
Readmissions, n (%)	Number of hospital patient readmissions within 30 days of discharge
Length of hospitalizations, days	Cumulative number of inpatient days
Outpatient visits	Number of encounters in outpatient settings
ER visits	Number of emergency room visits
Rehabilitation and PT visits	Number of encounters in rehabilitation facility and physical therapy visits
Filled prescriptions	Number of prescription medication claims

Table S3. Pain Medications by Class

**Analgesics/Antipyretics**	
acetaminophen	acetaminophen/atropine sulfate/pamabrom
acetaminophen/benzocaine	acetaminophen/bromelains
acetaminophen/caff/dihydrocod	acetaminophen/caffeine
acetaminophen/caffeine/calcium carbonate/glycine	acetaminophen/caffeine/isometheptene mucate
acetaminophen/caffeine/pamabrom/pyridoxine	acetaminophen/caffeine/pheniramine/phenylephrine
acetaminophen/caffeine/phenylephrine hydrochloride	acetaminophen/caffeine/phenyltoloxamine citrate
acetaminophen/caffeine/pseudoephedrine hydrochlori	acetaminophen/caffeine/salicylamide
acetaminophen/calcium carb	acetaminophen/calcium carbonate
acetaminophen/calcium/caf/glyc	acetaminophen/chlorpheniramine
acetaminophen/chlorpheniramine maleate	acetaminophen/chlorpheniramine/codeine/guaifenesin
acetaminophen/chlorpheniramine/dextromethorphan/gu	acetaminophen/citric acid/sodium bicarbonate
acetaminophen/d-brompheniramin	acetaminophen/dextromethorphan
acetaminophen/dextromethorphan hydrobromide	acetaminophen/dextromethorphan/guaifenesin/phenyle
acetaminophen/dp-hydram hcl	acetaminophen/dp-hydramine
acetaminophen/guaifenesin	acetaminophen/mag sal/pamabrom
acetaminophen/pamabrom	acetaminophen/pamabrom/pyridoxine hydrochloride
acetaminophen/pamabrom/vit b6	acetaminophen/pamabrom/vit e
acetaminophen/phenylephrine hydrochloride	acetaminophen/phenylpropanolamine hydrochloride
acetaminophen/phenyltolox/caff	acetaminophen/phenyltoloxamine
acetaminophen/phenyltoloxamine citrate	acetaminophen/pseudoephedrine hydrochloride
acetaminophen/pyril mal	acetaminophen/pyrilam/pamabrom
acetaminophen/salicylamide	acetaminophen/sodium bicarbonate
acetaminophen;acetaminophen	acetic acid/antipyrine/benzocaine/policosanol
acetic acid/salicylic acid	acetylcarnitine hydrochloride/arginine hcl/choline
acetylcarnitine hydrochloride/choline bitartrate/d	acetylcarnitine/choline bitartrate/cocoa extract/d
acetylcarnitine/choline bitartrate/cocoa extract/g	acetylcholine bromide
acetylcholine chloride	acetylcholine iodide
acetylcysteine/vit c/biotin/ca/choline/collagen, b	albuterol sulfate/ipratropium bromide
alcohol/salicylic acid	alfalfa/aminobenzoic acid/biotin/choline bitartrat
aloe/vitamin e/salicylic acid	alum/camphor/menthol/phenol/salicylic acid
aminobenzoic acid/bioflavonoid/biotin/ca/choline/c	aminobenzoic acid/biotin/ca pantothenate/choline b
aminobenzoic acid/biotin/ca pantothenate/choline d	aminobenzoic acid/biotin/ca pantothenate/choline/f
aminobenzoic acid/biotin/ca pantothenate/choline/v	aminobenzoic acid/biotin/choline bitartrate/folic
aminobenzoic acid/biotin/choline bitartrate/vit b1	aminobenzoic acid/vit c/betaine hcl/biotin/choline
aminobenzoic acid/vit c/biotin/choline/vit b12/fol	aminosalicylic acid
amlodipine besylate;arginine/caff/choline bitartra	antipyrine
antipyrine/benzocain/gly/zinc	antipyrine/benzocaine
antipyrine/benzocaine/gly/urea	antipyrine/benzocaine/glycerin
antipyrine/benzocaine/phenylephrine hydrochloride	antipyrine/benzocaine/policosanol
antipyrine/pyr. mal./sod. cap.	apap/caff/phenyltoloxamine cit/salicylamide
apap/caff/ppa hcl/salicylamide	apap/chlorpheniramine mal/salicylamide
apap/cpm/dm hydrobrom/phenyleph hcl/salicylamide	apap/cpm/phenyleph hcl/salicylamide
apap/phenyltoloxamine cit/salicylamide	arginine hcl/bromelains/caff/choline bitartrate/de
arginine/caff/choline bitartrate/cinnamon bark/coc	arginine/caff/choline bitartrate/cinnamon/cocoa ex
arginine/caff/choline bitartrate/cinnamon/cocoa/cy	arginine/choline bitartrate/cinnamon bark ext/gaba

arginine/choline bitartrate/cinnamon bark/cocoa/ga	arginine/choline bitartrate/cinnamon/cocoa extract
benzocaine/chloroxylenol/resorcinol/salicylic acid	benzocaine/salicylic acid
benzoic acid/camphor/methyl sal/salicylic acid	benzoic acid/salicylic acid
benzoyl peroxide;salicylic acid;salicylic acid	benzoyl peroxide;salicylic acid;salicylic acid;sun
benzoyl peroxide;salicylic acid;vitamin e	biotin/ca pantothenate/choline/cr/cu/fe/folic acid
biotin/ca/cholecalciferol/choline/cr/cu/fe/folic a	biotin/ca/choline/cr/cu/fe/folic acid/iodine/mg/mo
biotin/ca/choline/cu/fe/folic acid/iodine/mg/phosp	biotin/cholecalciferol/choline/vit b6;docosahexaen
biotin/choline/inositol/iodine/vit a/vit b12/vit b	bismuth/ca.carb./opium
bismuth/kaolin/pectin/opium	boric acid/salicylic acid/tannic acid
boron/ca pantothenate/choline/cu/inositol/se/vit a	brompheniramine/acetaminophen
bupropion hcl;caff/choline bitartrate/cocoa extrac	bupropion hcl;choline bitartrate/cocoa extract/glu
butalb/acetaminophen/caffeine	ca pantothenate/choline/fe/methionine/vitamin b3/v
ca pantothenate/choline/vit b12/folic acid/iodine/	ca/cholecalciferol/choline/cu/fe/folic acid/iodine
ca/cholecalciferol/choline/fe/folic acid/iodine/vi	ca/choline/vit b12/folic acid/vit b6/vit b2
caff/choline bitartrate/cocoa extract/glutamic aci	caff/phenolphthalein/ppa hcl/salicylamide
caff/ppa hcl/salicylamide	caffeine/choline/aa 11/hc130
caffeine/choline/aa 12/herb131	caffeine/choline/aa no.7/hb125
caffeine/potassium salicylate/salicylamide	calcium/choline/chromium/inositol/lecithin
calcium/choline/inositol	camphor/menthol/phenol/salicylic acid
cantharidin/podophyllum/salicylic acid	chlorphen/dm/acetaminophen/gg
cholecalciferol/choline/docosahexaenoic acid/vit b	choline
choline bit/aa 10/gaba/herb129	choline bit/amino acid7/hrb125
choline bit/calcium/b12/b6/gnk	choline bit/vit b comp/folic
choline bit/vit b12/vit b6/gnk	choline bitartrate
choline bitartrate/cocoa extract/glutamic acid/gra	choline bitartrate/d-panthenol
choline bitartrate/folic acid/inositol/methionine	choline bitartrate/glutamic acid/histidine/serine,
choline bitartrate/inositol	choline bitartrate/vit b12/inositol/vit b6/vit e
choline c 11	choline chloride
choline chloride/inositol/methionine	choline cl/vit b12/inositol/methionine
choline dihydrogen citrate	choline/cr picolinate/grapefruit/inositol/lecithin
choline/cyanocobalamin/ginkgo biloba/pyridoxine	choline/docosahexaenoic acid/eicosapentaenoic acid
choline/inositol	choline/vit b12/iodine/fe/mg/mn/niacinamide/vit b5
choline/vitamin b complex	coal tar/resorcinol/salicylic acid
coal tar/salicylic acid	coal tar/salicylic acid/sulfur
cod/salicylamide/apap	collodion/lactic acid/salicylic acid
cpm/pe/dm/acetaminophen/guaifn	d-methorphan/pe/acetaminophen
dezocine	diphenhyd/pe/acetaminophen/gg
dipyrone	dm/acetaminophen/diphenhydramn
dm/acetaminophen/doxylamine	dm/pe/acetaminophen/chlorphenr
dm/pe/acetaminophen/doxylamine	dm/ppa/acetaminophen/chlorphen
dm/ppa/acetaminophen/pyril	dm/pseudoephed/acetaminophen
dm/salicylamide/cpm/caffeine	docosahexaenoic acid;vit c/biotin/ca/choline/cu/vi
doxycycline;octinoxate/zinc oxide;salicylic acid	doxylam/pe/dm/acetaminophen/gg
fenofibric acid (choline)	fluorouracil;salicylic acid;cream base
gg/hydrocod bit/ppa hcl/salicylamide	guaifen/pe/acetaminophen/caff
guaifen/ppa/acetaminophen/cp	guaifenesin/acetaminophen
guaifenesin/dm/acetaminophen	guaifenesin/ppa/acetaminophen
hc;salicylic acid/sulfur;shampoo, multi ingredient	hydrochlorides of opium

hydrocortisone/salicylic acid	hydrocortisone;salicylic acid/sulfur
inosi/choline bit/vit b comp	inosi/choline bit/vit bcomp,c
inosit/choline/bcomp/niacin/b5	inositol/choline bitartrate
inositol/choline/biofla/vitb,c	inositol/choline/glutamine
inositol/choline/multivitamin	inositol/choline/vit b comp
iodine/salicylic acid	ipratropium bromide
ipratropium/albuterol sulfate	iron/inositol/choline
isomethepten/caf/acetaminophen	lactic acid/resorcinol/salicylic acid
metformin/aa 7/herb125/choline	methacholine chloride
methionine/inositol/choline	methoxyflurane
methyl salicylate/salicylic acid	methyl salicylate/salicylic acid/tolnaftate
mv-mn/fa/inosi/choline/bioflav	opium
opium tincture	opium/belladonna alkaloids
opium/bismuth/pectin/zn.phenol	opium/chlorphen/apap/phenylpro
opium/ipecac/caf/asp	opium/kaolin/pectin/bellad alk
opium/pectin/homatropine	p-ephed hcl/acetaminophen
p-ephed/acetaminophen/cromolyn	p-ephed/acetaminophen/doxylamn
pe/hydrocod/acetaminophen/cpm	pe/salicylmd/acetaminophen/cpm
petrolatum, white/salicylic acid/urea	ph-p-choline/dha/vitamin e/gnk
phenacetin	phenylephrine hcl/antipyrine
phosphatidyl choline	phosphatidyl choline/phosphatidylethanolamine/phos
phosphatidyl choline/vitamin b complex	phosphatidylcholine
ppa hcl/acetaminophen	ppa hcl/salicylamide/caffeine
ppa/acetaminophen	ppa/acetaminophen/br-phenir
ppa/acetaminophen/chlorphenir	ppa/acetaminophen/cpm/caffeine
ppa/acetaminophen/p-tlox/cpm	pseudoephed/acetaminophen/brom
pseudoephed/acetaminophen/cpm	pseudoephed/acetaminophen/d-cp
pseudoephedrine/acetaminophen	pyrilam/chlophed/acetaminophen
sal-amide/acetaminophen/p-tlox	salicylamide
salicylamide/acetaminophen	salicylamide/apap/phenyltolox
salicylamide/methyl nicotinate	salicylic acid
salicylic acid (alcohol & pg)	salicylic acid/alc/prop gly
salicylic acid/alcohol	salicylic acid/ammon lact/aloe
salicylic acid/ammoniated hg	salicylic acid/benzalk chl
salicylic acid/benzoic acid	salicylic acid/benzoyl peroxid
salicylic acid/boric acid	salicylic acid/ceramide comb 1
salicylic acid/coal tar	salicylic acid/coal tar/allant
salicylic acid/coal tar/menth	salicylic acid/coal tar/sulfur
salicylic acid/collodion, flex	salicylic acid/foot cushion
salicylic acid/glycerin	salicylic acid/isoprop.alcohol
salicylic acid/lactic acid	salicylic acid/lactic acid/c-t
salicylic acid/lactic acid/phe	salicylic acid/pyrithione zinc
salicylic acid/soap/sulfur	salicylic acid/sodium thiosulfate
salicylic acid/sulfacetamide	salicylic acid/sulfur
salicylic acid/undecylenic acid	salicylic acid/urea
salicylic acid/witch hazel	salicylic acid;cleanser
saliva collection/ibuprofen	sulfosalicylic acid

sulfosalicylic acid dihydrate	tiotropium br/olodaterol hcl
tiotropium bromide	triprolidine/pse/acetaminophen
vit b comp/niacin/b12/choline	vit b/folic/choline/inos/herbs
vit c/betaine/bioflavonoid/biotin/ca/choline/cr/cu	vit c/bioflavonoid/ca pantothenate/choline/vit b12
vit c/bioflavonoid/ca phos, dibasic/choline bitart	vit c/bioflavonoid/ca/choline/vit b12/inositol/nia
vit c/bioflavonoid/ca/choline/vit b12/inositol/vit	vit c/biotin/boron/ca/cholecalciferol/choline/cr/c
vit c/biotin/boron/ca/choline/cr/cu/vit b12/folic	vit c/biotin/boron/cholecalciferol/choline/cr/vit
vit c/biotin/ca/cholecalciferol/choline/cr/cu/vit	vit c/biotin/ca/cholecalciferol/choline/cu/vit b12
vit c/biotin/cholecalciferol/choline cl/cr/cu/foli	vit c/biotin/cholecalciferol/choline/vit b12/folic
vit c/biotin/choline bitartrate/cysteine/folic aci	vit c/biotin/choline/vit b12/ergocalciferol/inosit
vit c/biotin/choline/vit b12/folic acid/inositol/v	vit c/ca/ca carbonate/cholecalciferol/choline/vit
vit c/ca/cholecalciferol/choline/vit b12/folic aci	vit c/cholecalciferol/choline/choline alfoscerate/
vit c/cholecalciferol/choline/vit b12/docosahexaen	vit c/choline/vit b12/docosahexaenoic acid/eicosap
ziconotide acetate	zinc/salicylic acid/castor oil

**NSAIDs/COX-2 Inhibitors**	
acetaminophen/aluminum hydroxide/aspirin/caffeine	acetaminophen/aspirin
acetaminophen/aspirin, buffered	acetaminophen/aspirin/caffeine
acetaminophen/aspirin/caffeine/salicylamide	acetaminophen/aspirin/diphenhydramine citrate
acetaminophen/aspirin/salicylamide	acetaminophen/caffeine/magnesium salicylate
acetaminophen/magnesium salicylate	acetaminophen/magnesium salicylate/pamabrom
acetaminophen;ibuprofen	adapalene;diclofenac na;tamoxifen citrate;cream ba
al hydroxide/aspirin/ca carbonate/mg hydroxide	aluminum hydroxide/aspirin/magnesium hydroxide
amlodipine besylate/celecoxib	antipyrine/caffeine/sodium salicylate
apap/asa/pyril	apap;ibuprofen;simethicone;zn oxide;cleanser
asa/acetaminophen/caffeine/cal	asa/acetaminophen/caffeine/pot
asa/acetaminophen/mag/alh/caff	asa/apap/cal cb/caffein/vit b1
asa/caff/cinnamedrine	asa/cal carb/glycine/vit b1/vit b6
asa/calcium carb/mag/aluminum	asa/dp-hydramine/sod bicarb/ca
asa/mag hydrox/aluminum hydrox	asa/mag hydrox/aluminum/caff
asa/phenacet/caffeine/allobarb	asa/sal-amide/apap/al hydrox
asa/salicylam/acetaminoph/caff	aspirin
aspirin(calc&mg)/pravastatin	aspirin, buffered
aspirin/acetaminophen	aspirin/acetaminophen/caffeine
aspirin/acetaminophen/cal carb	aspirin/acetaminophen/phenacet
aspirin/apap/al hydrox/caffein	aspirin/brompheniramine mal/ppa hcl
aspirin/butalbital	aspirin/butalbital/caffeine
aspirin/ca carbonate/mg carbonate/mg oxide	aspirin/caff/mg carbonate/magnesium sal
aspirin/caffeine	aspirin/caffeine/dihydrocodein
aspirin/caffeine/magnesium salicylate	aspirin/caffeine/phenacetin
aspirin/caffeine/salicylamide	aspirin/calcium carbonate
aspirin/calcium carbonate/mag	aspirin/chlor-mal
aspirin/chlorpheniramine mal/dm hydrobrom/phenylep	aspirin/chlorpheniramine mal/dm hydrobrom/ppa hcl
aspirin/chlorpheniramine mal/phenyleph tar	aspirin/chlorpheniramine mal/ppa hcl
aspirin/chlorpheniramine mal/pse hcl	aspirin/citric acid/sodium bicarbonate
aspirin/dihydroxyaluminum aminoacetate/mg carbonat	aspirin/diphenhydramine citrat
aspirin/diphenhydramine citrate	aspirin/diphenhydramine hcl
aspirin/diphenhydramine hydrochloride	aspirin/dipyridamole

aspirin/dm hydrobrom/doxylamine succ/phenyleph tar	aspirin/dm hydrobrom/doxylamine succ/ppa hcl
aspirin/mag carb/aluminum	aspirin/mag hydrox/al hydrox
aspirin/magnesium oxide	aspirin/omeprazole
aspirin/pheniramine/phenylpropanolamine/pyrilamine	aspirin/phenylpropanolamine
aspirin/phenylpropanolamine bitartrate	aspirin/phenylpropanolamine hydrochloride
aspirin/phenyltoloxamine citrate	aspirin/pseudoephedrine hydrochloride
aspirin/sal-amide/apap/caffein	aspirin/salicylamide/caffeine
aspirin/salicylsalicylic acid	aspirin/sod bicarb/citric acid
aspirin;clopidogrel hydrogen sulfate	bacitracin zn/neomycin/polymyxin b sulf;ibuprofen
benzalkonium chloride;diclofenac sodium	benzoic acid/methenamine/sodium salicylate
bupivacaine hcl;isopropyl alc;ketorolac tromethami	bupivacaine hcl;ketorolac tromethamine;lido hcl
bupivacaine hcl;ketorolac tromethamine;lido hcl;po	bupivacaine/ketorolac/ketamine
butalbital/aspirin/caffeine	capsaicin/menthol/methyl sal;diclofenac na
capsaicin/menthol/methyl salicylate;meloxicam	capsaicin/menthol/methyl salicylate;naproxen
capsaicin;diclofenac na;ranitidine hcl	capsaicin;diclofenac sodium
capsaicin;diclofenac sodium;omeprazole	celecoxib
celecoxib/capsai/m-sal/menthol	celecoxib/capsaicin/menthol
celecoxib/lidocaine/menthol	chlorphen/phenyleph/dm/aspirin
chlorphen/phenyleph/ibuprofen	chlorphen/pseudoeph/ibuprofen
chlorphenir/phenyleph/aspirin	chlorpheniramine mal/ibuprofen/phenyleph hcl
chlorpheniramine-ppa-aspirin	chol sal/magnesium salicylate
choline magnesium trisalicylate	choline salicyl/mag salicylate
choline salicylate	cinnamedrine hcl/asa/caffeine
cod/asa/salicylmd/acetamin/caf	dextromethorphan/ppa/asa/bpm
dextromethorphan/ppa/asa/cpm	diclofenac epolamine
diclofenac na;folic acid/methylcobalamin/vit b6	diclofenac na;lido/prilocaine;ranitidine hcl
diclofenac potassium	diclofenac sod/trolamine salic
diclofenac sodium	diclofenac sodium, micronized
diclofenac sodium/capsaicin	diclofenac sodium/lidocaine
diclofenac sodium/menthol	diclofenac sodium/misoprostol
diclofenac sodium;cream base	diclofenac sodium;isopropyl alcohol;metaxalone
diclofenac sodium;menthol	diclofenac submicronized
diclofenac/benzalkonium chlor	diclofenac/capsicum oleoresin
diclofenac/hyaluronate/niacin	diclofenac/kinesiology tape
diclofenac/lido/me-sal/camphor	diclofenac/lidocaine/tape
diclofenac/me-salic/menth/camp	diclofenac/menthol/camphor
diclofenac/menthol/tape	diclofenac/met salicyl/menthol
diclofenac/silicone, adhesive	diethylene glycol monoethyl ether;ketoprofen;cream
diflunisal	diphenhydramine citrate/ibuprofen
diphenhydramine hydrochloride/naproxen sodium	dovers/aspirin/caf
doxylamine/phenylep/dm/aspirin	ephedrine/asa/acetan/caffeine
esomeprazole magnesium/naproxen	esomeprazole magnesium;ibuprofen;lidocaine/menthol
etodolac	etodolac/capsaicin/me-sal/ment
fenoprofen calcium	fenoprofen calcium, dihydrate
flurbiprofen	flurbiprofen sodium
flurbiprofen, micronized	ibuprofen
ibuprofen lysine/pf	ibuprofen/caff/b1/b2/b6/b12

ibuprofen/capsai/m-sal/menthol	ibuprofen/diet. supp 11
ibuprofen/diphenhydramine cit	ibuprofen/diphenhydramine hcl
ibuprofen/famotidine	ibuprofen/irr.count-irrit.no.2
ibuprofen/phenylephrine hcl	ibuprofen/pseudoephedrine hcl
ibuprofen/pseudoephedrine hydrochloride	ibuprofen;cream, multi ingredient
ibuprofen;medical food	ibuprofen;prasterone
indomethacin	indomethacin sodium
indomethacin, submicronized	isopropyl alc;ketorolac tromethamine;lido hcl;pov
ketamine hcl;ketoprofen;lecithin;lido hcl;cream ba	ketoprofen
ketoprofen, micronized	ketoprofen;cream, multi ingredient
ketoprofen;lecithin;cream base	ketoprofen;lecithin;lido hcl;cream base
ketorolac tromethamine	ketorolac tromethamine/pf
ketorolac/norflurane/hfc 245fa	ketorolac/phenylephrine
lansoprazole/naproxen	mag salicylat/acetaminoph/caff
mag salicylate/acetaminophen	mag salicylate/diphenhydramine
mag salicylate/phenyltolox	mag salicylate/phenyltolx
magnesium salicylate	magnesium salicylate/caffeine
magnesium salicylate/phenyltoloxamine citrate	meclofenamate sodium
mefenamic acid	meloxicam
meloxicam, submicronized	meloxicam/irrit.cntr-irr cmb 2
meloxicam;medical food	menthol;naproxen sodium
methacholine/methyl salicylate	methenamine/sodium salicylate
nabumetone	nabumetone, micronized
nabumetone/capsai/m-salicy/men	naproxen
naproxen sod/diphenhydramine	naproxen sodium
naproxen sodium/menthol	naproxen sodium/pseudoephedrin
naproxen sodium/pseudoephedrine hydrochloride	naproxen/capsai/menthol/me-sal
naproxen/capsaicin/menthol	naproxen/diet. supp 11
naproxen/esomeprazole mag	naproxen/irritant cntr-irrit 2
naproxen;cream, multi ingredient	naproxen;medical food
opium/aspirin/caffeine	opium/aspirin/caffeine/camphor
oxaprozin	phenyleph hcl/asa/cp/caffein
phenylephrine hcl/aspirin	phenylephrine/ketorolac
piroxicam	piroxicam/diet. supp 11
piroxicam;medical food	ppa bit/aspirin
ppa bit/aspirin/chlorphenir	ppa hcl/aspirin/acetaminophen
ppa hcl/aspirin/br-phenir	ppa hcl/aspirin/chlorphenir
ppa/asa/chlorphenir/caffeine	ppa/aspirin/chlorpheniramine
ppa/aspirin/diphenhydramine	pseudo/aspirin/caffeine
pseudoephed/asa/chlorphenir	pseudoephedrine hcl/aspirin
rofecoxib	ropivacaine/clonidin/ketorolac
ropivacaine/ketorolac/ketamine	salsalate
sodium salicylate	sodium salicylate/sulfur
sodium thiosalicylate	sulindac
sumatriptan succ/naproxen sod	tolmetin sodium
valdecoxib	

Intra-articular Corticosteroids / Hyaluronic Acid

ammonia/etoh;lido hcl;pov iodine;triamcinolone ace	betamethasone
betamethasone ace,sod phos/wtr	betamethasone ace/betamethasone na phos
betamethasone ace/betamethasone na phos;bupivacain	betamethasone ace/betamethasone na phos;isopropyl
betamethasone acetate,sod phos	betamethasone sodium phosphate
bupivacaine hcl;lido hcl;methylprednisolone acetat	bupivacaine hcl;lido hcl;pov iodine;triamcinolone
bupivacaine hcl;methylprednisolone acetate;pov iod	bupivacaine hcl;pov iodine;triamcinolone acetonide
dexameth na phos/lido hcl	dexameth na phos;isopropyl alc;pov iodine
dexamethasone	dexamethasone ac, sod ph/water
dexamethasone ace/nacl,iso-osm	dexamethasone acetate
dexamethasone sodium phosphate	dexamethasone/lidocaine hcl
hyaluronate sodium	hyaluronate sodium, stabilized
hyaluronic acid	isopropyl alc;lido hcl;methylprednisolone acetate
isopropyl alc;lido hcl;methylprednisolone acetate;	isopropyl alc;lido hcl;pov iodine;triamcinolone ac
isopropyl alc;methylprednisolone acetate;pov iodin	isopropyl alc;pov iodine;triamcinolone acetonide
isopropyl alcohol;triamcinolone acetonide	lido hcl;pov iodine;triamcinolone acetonide
lidocaine hydrochloride/triamcinolone acetonide	lidocaine/prilocaine;triamcinolone acetonide
methylprednisolone	methylprednisolone acet-water
methylprednisolone acetate	methylprednisolone sod succ
methylprednisolone sodium succinate	triamcinolone acet/0.9%nacl/pf
triamcinolone aceton/0.9% nacl	triamcinolone acetonide
triamcinolone acetonide;cleanser;cleanser and mois	triamcinolone acetonide;cream, multi ingredient
triamcinolone dia/0.9% nacl/pf	triamcinolone diacet/0.9% nacl
triamcinolone diacetate	triamcinolone hexacetonide
triamcinolone/bupivacaine/nacl	

Tramadol

acetaminophen/tramadol hydrochloride	camphor;gabapentin;menthol;tramadol hydrochloride;
tramadol hcl	tramadol hcl/acetaminophen
tramadol hydrochloride	tramadol hydrochloride;cream, multi ingredient
tramadol hydrochloride;medical food	tramadol/dietary supp. no. 11
tramadol/glucosamine	

Non-tramadol Opioids

a.p.c. w/codeine	acetaminophen with codeine
acetaminophen/aspirin/caffeine/codeine phosphate	acetaminophen/aspirin/codeine phosphate
acetaminophen/butalbital/caffeine/hydrocodone	acetaminophen/butalbital/codeine phosphate
acetaminophen/caffeine/dihydrocodeine bitartrate	acetaminophen/codeine phosphate
acetaminophen/hydrocodone bitartrate	acetaminophen/meperidine hydrochloride
acetaminophen/oxycodone hydrochloride	acetaminophen/pentazocine hydrochloride
acetaminophen/propoxyphene hydrochloride	acetaminophen/propoxyphene napsylate
alfentanil hcl	alfentanil hydrochloride
ammonium chloride/codeine phosphate/guaifenesin	ammonium chloride/codeine/chlorpheniramine/guaifen
ammonium cl/chlorpheniramine mal/codeine phos/phen	ammonium cl/chlorpheniramine/codeine/phenylephrine
apap/aspirin/caff/codeine phos/salicylamide	apap/aspirin/caff/codeine/salicylamide
apap/butabarbital na/codeine phos	apap/butalbital/caff/codeine phos
apap/caff/codeine/salicylamide	apap/chlorpheniramine mal/codeine phos/phenyleph h
apap/codeine phos;medical food	aspirin (buffered)/codeine phosphate

aspirin/butalbital/caffeine/codeine phosphate	aspirin/caffein/dihydrocodeine
aspirin/caffeine/dihydrocodeine bitartrate	aspirin/caffeine/hydrocodone bitartrate
aspirin/caffeine/propoxyphene hydrochloride	aspirin/codeine phosphate
aspirin/hydrocodone bitartrate	aspirin/oxycodone hcl/oxycodone terephthalate
aspirin/propoxyphene hydrochloride	aspirin/propoxyphene napsylate
benzhydrocodone/acetaminophen	bismuth/kaolin/pectin/codeine
bpm/dihydrocodeine bitartrate/pse hcl	bromodiphenhydramine hcl/codeine phos
brompheniram/pe/dihydrocodeine	brompheniramine mal/codeine phos/ppa hcl
brompheniramine mal/codeine phos/pse hcl	brompheniramine tan/hydrocodone tannate/pse tan
brompheniramine/codeine phos	brompheniramine/p-eph/codeine
bupivacaine hcl/epi hcl/fentanyl citrate/na cl	bupivacaine hcl/fentanyl citrate/na cl
bupivacaine hcl/hydromorphone hydrochloride/na cl	bupivacaine hcl/morphine sulf/na cl
bupivacaine hcl/na cl/sufentanil citrate	bupivacaine/fentanyl/sodium chloride
buprenorphine	buprenorphine hcl
buprenorphine hcl/naloxone hcl	buprenorphine hydrochloride
buprenorphine/naloxone	butalbit/acetamin/caff/codeine
butorphanol tartrate	chlor-mal/codeine/acetaminophn
chlorcycli/pseudoephed/codeine	chlorcycliz/phenyleph/codeine
chlorcyclizine hcl/codeine ph	chlorcyclizine hcl/codeine phos/pse hcl
chlorcyclizine hydrochloride/codeine phosphate	chlorphen/pseudoephed/codeine
chlorpheniramine mal/codeine phos/gg/pse hcl	chlorpheniramine mal/codeine phos/phenyleph hcl/ki
chlorpheniramine mal/codeine phos/phenyleph hcl/pp	chlorpheniramine mal/codeine phos/ppa hcl
chlorpheniramine mal/codeine phos/pse hcl	chlorpheniramine mal/dihydrocodeine bitartrate/phe
chlorpheniramine mal/dihydrocodeine bitartrate/pse	chlorpheniramine maleate/codeine phosphate
chlorpheniramine maleate/hydrocodone bitartrate	chlorpheniramine polistirex/hydrocodone polistirex
chlorpheniramine/codeine phos	chlorpheniramine/codeine/guaif
chlorpheniramine/codeine/phenylephrine	chlorpheniramine/pe/codeine
cocillana w/codeine	codeine phos/acetaminophen
codeine phos/chlor-mal	codeine phos/gg/ppa hcl
codeine phos/gg/pse hcl	codeine phos/phenyleph hcl/promethazine hcl
codeine phos/phenyleph hcl/pyril mal	codeine phos/pse hcl/triprolidine hcl
codeine phosphate	codeine phosphate/apap
codeine phosphate/aspirin	codeine phosphate/br-dpha hcl
codeine phosphate/guaifenesin	codeine phosphate/iodinated glycerol
codeine phosphate/promethazine hydrochloride	codeine phosphate/pseudoephedrine hydrochloride
codeine phosphate/pyrilamine	codeine poli/chlorphenir polis
codeine sulfate	codeine/butalbital/asa/caffein
codeine/calcium iodide	codeine/dextromethorphan/guaifenesin/pseudoephedri
codeine/ephedrine/guaifenesin	codeine/guaifenesin/phenylephrine/phenylpropanolam
codeine/terpin hydrate	dexbrompheniramine/pse/codeine
dexchlorphen/phenyleph/codeine	dextrose/fentanyl citrate
dextrose/hydromorphone hydrochloride	dextrose/morphine sulfate
dihydrocodeine bitartrate	dihydrocodeine bitartrate/gg/pse hcl
dihydrocodeine bitartrate/phenyleph hcl	dihydrocodeine/apap/caffeine
dihydrocodeine/cpm/pseudoephed	dihydrocodeine/guaifenesin
diphenhydramin/pe/codeine phos	ethylmorphine hcl
fentanyl	fentanyl citrat/dextrose 5%/pf
fentanyl citrate	fentanyl citrate-0.9 % nacl/pf

fentanyl citrate/droperidol	fentanyl citrate/pf
fentanyl citrate/ropivacaine hcl/na cl	fentanyl citrate/sodium chloride
fentanyl hcl	fentanyl/bupivacaine/ns/pf
fentanyl/ropivacaine/ns/pf	fentanyl/ropivacaine/sodium chloride
guaifen/hydrocodone/br-phenir	guaifen/p-propanolamin/codeine
guaifenesin/codeine phos	guaifenesin/codeine phosphate
guaifenesin/hydrocodone	guaifenesin/hydrocodone bitartrate
homatropine methylbromide/hydrocodone bitartrate	hydrocodone bit/acetaminophen
hydrocodone bit/homatrop me-br	hydrocodone bitartrate
hydrocodone bitartrate/apap	hydrocodone bitartrate/aspirin
hydrocodone bitartrate/ibuprofen	hydrocodone bitartrate/potassium guaiacolsulfonate
hydrocodone tannate/chlor-tan	hydrocodone tannate/pseudoephedrine tannate
hydrocodone/acetam/diet.sup.11	hydrocodone/acetaminophen
hydrocodone/am. cl/antihist.	hydrocodone/asp/caf
hydrocodone/bromphen/pseudoeph	hydrocodone/carbinox/pseudoeph
hydrocodone/chlorphen p-stirex	hydrocodone/chlorpheniramine
hydrocodone/cpm/pseudoephed	hydrocodone/cpm/pseudoephed/gg
hydrocodone/homatropine	hydrocodone/ibuprofen
hydrocodone/k guaiacolsulfonate/pse hcl	hydrocodone/pheniramine/phenylephrine/ppa
hydrocodone/pseudoephed/guaif	hydromorphone hcl
hydromorphone hcl in 0.9% nacl	hydromorphone hcl in d5w/pf
hydromorphone hcl in water/pf	hydromorphone hcl/0.9% nacl/pf
hydromorphone hcl/guaifenesin	hydromorphone hcl/pf
hydromorphone hydrochloride	hydromorphone hydrochloride/ropivacaine hcl/na cl
hydromorphone hydrochloride/sodium chloride	hydromorphone/bupiv/0.9nacl/pf
hydromorphone/ropiv/sod chl/pf	ibuprofen/oxycodone hcl
ibuprofen/oxycodone hydrochloride	levorphanol tartrate
meperidine hcl	meperidine hcl in 0.9 % nacl
meperidine hcl in 0.9% nacl/pf	meperidine hcl/atropine
meperidine hcl/pf	meperidine hcl/prometh hcl
meperidine hcl/promethazine hcl	meperidine hydrochloride
meperidine hydrochloride/sodium chloride	methadone hcl
methadone hydrochloride	methadone hydrochloride/sodium chloride
methadone in 0.9 % sod.chlorid	morphine in iso sod chlorid/pf
morphine sulfate	morphine sulfate in 0.9 % nacl
morphine sulfate liposomal/pf	morphine sulfate/0.9% nacl/pf
morphine sulfate/atropine sulf	morphine sulfate/d5w
morphine sulfate/d5w/pf	morphine sulfate/naltrexone
morphine sulfate/naltrexone hydrochloride	morphine sulfate/pf
morphine sulfate/sodium chloride	nalbuphine hcl
nalbuphine hydrochloride	nalbuphine hydrochloride/sodium chloride
naloxone hydrochloride/pentazocine hydrochloride	oxycodone hcl
oxycodone hcl,terephth/aspirin	oxycodone hcl/acetaminophen
oxycodone hcl/aspirin	oxycodone hydrochloride
oxycodone myristate	oxycodone/aspirin
oxymorphone hcl	oxymorphone hydrochloride
p/ephed/codeine/acetaminoph/gg	papaverine/codeine
pe-codeine-acetaminophen-guaif	pe/codeine/acetaminophen/cpm

pe/codeine/pheniramine/sod cit	pe/hydrocodone/bromphen tannts
pe/hydrocodone/dexbromphenirmn	pe/hydrocodone/dexchlor tannts
pentazocine hcl/acetaminophen	pentazocine hcl/aspirin
pentazocine hcl/naloxone hcl	pentazocine lactate
phenyleph/dihydrocodeine/guaif	phenylephrine hcl/codeine
phenylephrine hcl/hydrocodone	phenylephrine/codeine/guaifen
phenylephrine/dhcodeine bt/cpm	phenylephrine/hydrocodone/bpm
phenylephrine/hydrocodone/cpm	phenylephrine/hydrocodone/d-cp
phenylephrine/hydrocodone/dpha	pot guaiaco/hydrocodone
pot. citrate/tolu/codeine	ppa/codeine/brompheniramine
ppa/hydrocodone bitartrate	promethazine hcl/codeine
promethazine/phenyleph/codeine	propoxyphene hcl
propoxyphene hcl/acetaminophen	propoxyphene hydrochloride
propoxyphene nap/acetaminophen	propoxyphene napsylate
propoxyphene/aspirin/caffeine	pseudoephed/codeine/guaifen
pseudoephed/codeine/triprolidn	pseudoephed/hydrocodone
pseudoephed/hydrocodone/tripro	pseudoephedrine hcl/codeine
pyril ma/pe/codeine phos	pyril mal/pseudoephed/codeine
pyrilam/phenyle/dihydrocodeine	remifentanil hcl
remifentanil hydrochloride	remifentanil hydrochloride/sodium chloride
remifentanil in 0.9 % nacl/pf	ropivacaine hcl/na cl/sufentanil citrate
sufentanil citrate	sufentanil citrate/pf
sufentanil/bupivacaine/ns/pf	tapentadol hcl
tapentadol hydrochloride	theoph/eph/guai/codeine
triprolidine/phenyleph/codeine	

Antidepressants

5-hydroxytryptophan (5-htp)	5hydroxytryptophan(oxitriptan)
amitriptyline hcl	amitriptyline hcl;clonidine hcl;flurbiprofen;gabap
amitriptyline hcl;cream, multi ingredient	amitriptyline hcl;diclofenac na;lido hcl;prilocain
amitriptyline hcl;medical food	amitriptyline hydrochloride
amitriptyline hydrochloride/perphenazine	amoxapine
bupropion hbr	bupropion hcl
bupropion hydrobromide	bupropion hydrochloride
bupropion hydrochloride/naltrexone hydrochloride	bupropion hydrochloride;medical food
bupropion/dietary supp. no.15	bupropion/dietary supp. no.16
chamomile/inositol/melatonin/tryptophan/valerian r	citalopram hydrobromide
clomipramine hcl	clomipramine hydrochloride
desipramine hcl	desipramine hydrochloride
desvenlafaxine	desvenlafaxine fumarate
desvenlafaxine succinate	doxepin hcl
doxepin hydrochloride	duloxetine hcl
duloxetine hydrochloride	duloxetine hydrochloride;lidocaine/menthol
escitalopram oxalate	esketamine hcl
fluoxetine	fluoxetine hcl
fluoxetine hcl;medical food	fluoxetine hydrochloride
fluoxetine hydrochloride/olanzapine	fluoxetine/dietary supp no.17
fluoxetine/dietary supp. no.8	fluvoxamine maleate

hydroxytryptophan	hydroxytryptophan/melatonin
hydroxytryptophan/mg/melatonin/vit b6/tryptophan	hydroxytryptophan/pyridoxine
imipramine hcl	imipramine hydrochloride
imipramine pamoate	isocarboxazid
levomilnacipran hcl	levomilnacipran hydrochloride
levomilnacipran hydrochloride;levomilnacipran hydr	maprotiline hcl
maprotiline hydrochloride	melatonin/tryptophan
milnacipran hcl	milnacipran hydrochloride
mirtazapine	naltrexone hcl/bupropion hcl
nefazodone hcl	nefazodone hydrochloride
nortriptyline hcl	nortriptyline hydrochloride
olanzapine/fluoxetine hcl	paroxetine hcl
paroxetine hydrochloride	paroxetine mesylate
perphenazine/amitriptyline hcl	phenelzine sulfate
protriptyline hcl	protriptyline hydrochloride
pyridoxine hcl/tryptophan	sertraline hcl
sertraline hydrochloride	tranylcypromine sulfate
trazodone hcl	trazodone hydrochloride
trazodone hydrochloride;medical food	trazodone/dietary supp. no.8
trimipramine maleate	tryptophan
venlafaxine hcl	venlafaxine hydrochloride
vilazodone hcl	vilazodone hydrochloride
vilazodone hydrochloride;vilazodone hydrochloride	vortioxetine hydrobromide

**Muscle Relaxants**	
acetaminophen/chlorzoxazone	amantadine hcl;amitriptyline hcl;cyclobenzaprine h
amantadine hcl;baclofen;bupivacaine hcl;cyclobenza	amantadine hcl;baclofen;diclofenac na;gabapentin;l
aspirin/caffeine/orphenadrine citrate	aspirin/carisoprodol
aspirin/carisoprodol/codeine phosphate	aspirin/methocarbamol
atracurium besylate	baclofen
baclofen;cream, multi ingredient	baclofen;cyclobenzaprine hcl;flurbiprofen;lido hcl
baclofen;flurbiprofen;lido hcl;cream, multi ingred	baclofen;gabapentin;ketoprofen;cream base
baclofen;gabapentin;ketoprofen;lido hcl;cream, mul	baclofen;suspension, multi ingredient
carisoprodol	carisoprodol/aspirin
carisoprodol/aspirin/codeine	carisoprodol/diet. supp 11
carisoprodol;medical food	chlormezanone
chlorzoxazone	chlorzoxazone/acetaminophen
cisatracurium besylate	cyclobenzaprine
cyclobenzaprine hcl	cyclobenzaprine hcl;cream, multi ingredient
cyclobenzaprine hcl;dexamethasone;flurbiprofen;gab	cyclobenzaprine hcl;diethylene glycol monoethyl et
cyclobenzaprine hcl;flurbiprofen;cream base	cyclobenzaprine hcl;medical food
cyclobenzaprine hydrochloride	cyclobenzaprine hydrochloride;gabapentin
cyclobenzaprine with msm	cyclobenzaprine/capsai/menthol
cyclobenzaprine/diet. supp. 11	cyclobenzaprine/irr cntr-irr 2
cyclobenzaprine/tens electrode	cyclobenzaprine/tens unit/elec
dantrolene sodium	doxacurium chloride
hexafluorenium	mephenesin

methocarbamol	methocarbamol/aspirin
mivacurium chloride	mivacurium chloride/d5w
orphenadrine	orphenadrine citrate
orphenadrine/acetaminophen	orphenadrine/aspirin/caffeine
pancuronium bromide	pipecuronium bromide
rocuronium bromide	sodium chloride/vecuronium bromide
succinylcholine chloride	succinylcholine in 0.9% nacl
succinylcholine in 0.9%nacl/pf	succinylcholine/sod clr,iso/pf
tizanidine hcl	tizanidine hydrochloride
tizanidine/irritant cntr-irrt2	tubocurarine chloride
vecuronium bromide	vecuronium bromide/water
**Benzodiazepines**	
alprazolam	alprazolam/dietary suppl no.17
alprazolam;medical food	amitriptyline hydrochloride/chlordiazepoxide
amitriptyline/chlordiazepoxide	chlordiazepoxide
chlordiazepoxide hcl	chlordiazepoxide hydrochloride
chlordiazepoxide hydrochloride/clidinium bromide	chlordiazepoxide/clidinium br
chlordiazepoxide/esterified estrogens	chlordiazepoxide/methylscopola
clobazam	clonazepam
clorazepate dipotassium	dextrose/lorazepam
dextrose/midazolam hydrochloride	diazepam
diazepam (in soybean oil)	diazepam/soybean oil
diazepam;lubricant	diazepam;medical food
estazolam	flurazepam hcl
flurazepam hydrochloride	lorazepam
lorazepam in 5 % dextrose	lorazepam/0.9% sodium chloride
lorazepam/sodium chloride	midazolam
midazolam hcl	midazolam hcl in 0.9 % nacl/pf
midazolam hcl/pf	midazolam hydrochloride
midazolam hydrochloride/sodium chloride	midazolam in 0.9 % sod.chlorid
midazolam in 5 % dextrose	midazolam in 5 % dextrose/pf
midazolam/ketamine/ondansetron	oxazepam
quazepam	temazepam
temazepam/dietary sup. no.8	temazepam;medical food
triazolam	
**Anxiolytics/Sedatives/Hypnotics**	
acetaminophen/butabarbital sodium	acetaminophen/butalbital
acetaminophen/butalbital/caffeine	acetaminophen/caffeine/pyrilamine maleate
acetaminophen/diphenhydramine	acetaminophen/diphenhydramine citrate
acetaminophen/diphenhydramine hydrochloride	acetaminophen/melatonin
acetaminophen/pamabrom/pyrilamine maleate	acetaminophen/pyrilamine mal
acetaminophen/pyrilamine maleate	acetaminophen/pyrilamine/caff
acetylcarbromal/acetaminophen	allantoin/diphenhydramine hydrochloride
aminoglutethimide	aminophylline/amobarbital sodium/ephedrine
amobarbital	amobarbital sod/secobarbital
amobarbital sodium	amobarbital sodium/secobarbital sodium

antipyrine/phenylephrine/pyrilamine	apap/cpm/diphenhydramine hcl/phenyleph hcl
apap/cpm/pse hcl;apap/diphenhydramine hcl/pse hcl	apap/dichloralphenazone/isometheptene mucate
apap/diphenhydramine hcl/gg/phenyleph hcl	apap/diphenhydramine hydrochloride/phenyleph hcl
apap/diphenhydramine hydrochloride/phenyleph hcl;a	apap/diphenhydramine hydrochloride/phenyleph hcl;d
apap/diphenhydramine hydrochloride/pse hcl	apap/diphenhydramine hydrochloride/pse hcl;apap/dm
apap/dm hydrobrom/diphenhydramine	apap/dm hydrobrom/diphenhydramine hcl/phenyleph hc
apap/dm hydrobrom/diphenhydramine hydrochloride/ps	apap;apap/diphenhydramine hydrochloride
aprobarbital	atenolol/scopolamine
atropine sulf/hyoscyamine sulf/scopolamine hcl	atropine/scopolamine/hyoscy
barbital	barbital sodium
barbital/hyoscy/passifl/valeri	bellad alk/butabarbital sodium
belladonna alkaloids/butabarbital	benzethonium cl/diphenhydramine hydrochloride/zn a
benzocaine/calamine/diphenhydramine hydrochloride/	benzocaine/pyrilamine maleate
benzocaine/pyrilamine maleate/zinc oxide	benzocaine/pyrilamine/zinc oxide
black cohosh root/hop ext/melatonin/valerian root	bpm/diphenhydramine hcl/phenyleph hcl
bromelains/glycine/lemon balm/melatonin/mg/theanin	bromelains/melatonin/herbal233
brompheniramine mal/diphenhydramine hydrochloride	buspirone hcl
buspirone hydrochloride	butabarbital sodium
butabarbital/ephedrine/guaifenesin/theophylline	butabarbital/gg/pseudoephedrine/theoph
butabarbital/hyoscyamine/phenazopyridine	butalbital
butalbital/acetaminophen	ca/glutamine/inositol/melatonin/vitamin b3/passion
calamine/camphor/diphenhydramine hydrochloride	calamine/diphenhydramine hydrochloride
calcium phosphate/melatonin	calcium/soy/cohosh/melatonin
camphor/diphenhydramine hydrochloride/zinc oxide	carbetapentane tannate/diphenhydramine tannate
carbetapentane tannate/pyrilamine tannate	carbinoxamine mal/methscopolamine nitrate/pse hcl
carbinoxamine mal/methscopolamine/pse hcl	carbromal
chamomile/gaba/holy basil/hop/lemon balm/melatonin	chamomile/inositol/melatonin/vitamin b3/vit b6/try
chamomile/lavender/lemon balm/melatonin	chamomile/lavender/lemon balm/melatonin/soy isofla
chlorpheniramine mal/methscopolamine nitrate/pheny	chlorpheniramine mal/methscopolamine nitrate/pse h
chlorpheniramine mal/methscopolamine nitrate;chlor	chlorpheniramine mal/methscopolamine nitrate;meths
chlorpheniramine mal/phenylephrine/ppa/pyrilamine	chlorpheniramine maleate/methscopolamine nitrate
chlorpheniramine maleate/scopolamine methonitrate	chlorpheniramine/phenylephrine/ppa/pyrilamine
cpm tan/methscopolamine nitrate/phenyleph tan	cpm/dm hydrobrom/methscopolamine nitrate
cyanocobalamin/folic acid/melatonin	dehydroepiandrosterone/melatonin/pregnenolone
dexmedetomidine hcl	dexmedetomidine hydrochloride
dexmedetomidine hydrochloride/sodium chloride	dexmedetomidine in 0.9 % nacl
dexmedetomidine in dextrose 5%	dextromethorphan hydrobromide/pyrilamine maleate
dextromethorphan/pe/pyrilamine	dichloralphenazone
diphenhydramine citrate	diphenhydramine citrate/pse hcl
diphenhydramine hcl	diphenhydramine hcl/allantoin
diphenhydramine hcl/benzyl alc	diphenhydramine hcl/calamine
diphenhydramine hcl/camph/cala	diphenhydramine hcl/hydrocod bit/phenyleph hcl
diphenhydramine hcl/menthol	diphenhydramine hcl/zinc acet
diphenhydramine hydrochloride	diphenhydramine hydrochloride/menthol
diphenhydramine hydrochloride/phenyleph hcl	diphenhydramine hydrochloride/phenyleph hcl;cough/
diphenhydramine hydrochloride/phenyleph hcl;dm hyd	diphenhydramine hydrochloride/phenyleph hcl;phenyl
diphenhydramine hydrochloride/pramoxine hcl	diphenhydramine hydrochloride/pse hcl

diphenhydramine hydrochloride/resorcinol	diphenhydramine hydrochloride/tripelennamine hcl
diphenhydramine hydrochloride/zinc acetate	diphenhydramine hydrochloride/zinc oxide
diphenhydramine hydrochloride;hc;nystatin;mouthwas	diphenhydramine hydrochloride;hc;nystatin;tetracyc
diphenhydramine hydrochloride;lido hcl;mouthwash	diphenhydramine hydrochloride;lido hcl;nystatin;mo
diphenhydramine hydrochloride;prednisone	diphenhydramine tannate
diphenhydramine tannate/phenylephrine tannate	diphenhydramine/0.9 % sod.chlr
diphenhydramine/camphor/menth.	diphenhydramine/dm
diphenhydramine/hydrocortisone	diphenhydramine/niacin/apap
diphenhydramine/phenylephr/dm	dm hydrobrom/diphenhydramine hcl/phenyleph hcl
dm tan/diphenhydramine tannate/phenyleph tan	dm,p-ephed,pyrilamine tannates
dm-pe-pyrilamine tannates	dm/pe/acetaminophen/pyrilamine
eph sulf/hydroxyzine hydrochloride/theoph	ephedrine w/amobarbital
ephedrine/secobarbital	eszopiclone
ethchlorvynol	etomidate
fospropofol disodium	gamma aminobutyric acid/melatonin/valerian
glutethimide	guaifenesin/ephedrine/pyrilamine/theophylline
halazepam	hydroxyzine hcl
hydroxyzine hydrochloride	hydroxyzine pamoate
melatonin	melatonin/5htp/valer/fa/diet28
melatonin/chamomile flower	melatonin/gaba/herbal no.183
melatonin/gaba/valerian	melatonin/herbal comb. no.184
melatonin/herbal no.233	melatonin/kava root/griffonia
melatonin/lemon balm leaf extr	melatonin/pyridoxal phosphate
melatonin/pyridoxine	melatonin/pyridoxine hcl (b6)
melatonin/theanine	melatonin/val/chamomile flower
melatonin/valerian root	methohexital in water/pf
methohexital sodium	methscopolamine bromide
methscopolamine bromide;lactobacillus combination	methscopolamine nitrate
methscopolamine nitrate/phenyleph hcl;cough/cold c	methscopolamine nitrate/pse hcl
methscopolamine nitrate/pse hcl;cough/cold combina	methylscopolamine nitrate
methyprylon	oxyphencyclimine/hydroxyzine
pe/pe & pyrilamine tannate/cpm	pentobarbital
pentobarbital sodium	phenylephrine hcl/scopolamine
phenylephrine hydrochloride/pyrilamine maleate	phenylephrine/diphenhydramine
phenylephrine/methscopolamine	phenylephrine/pyrilamine
phenylephrine/pyrilamine ma/cp	phenylephrine/pyrilamine tan
ppa hcl/chlor-mal/scopolamine	ppa/pyrilamine maleate/phenir
ppa/pyrilamine/phenyltolox/pnm	pramoxine hcl/diphenhydramine
prazepam	propiomazine hcl
propiomazine hydrochloride	propiomazine maleate
propofol	propofol/pf
propranolol/scopolamine	pseudoephed tan/pyrilamine tan
pseudoephed/diphenhydramine	pyrilamine mal/dexbromphen tan
pyrilamine mal/pseudophed hcl	pyrilamine maleate
pyrilamine tannate	pyrilamine/benzocaine/zinc ox
pyrilamine/chlophedianol	pyrilamine/dextromethorphan hb
pyrilamine/dimethyl/polysilox	pyrilamine/etoh/benzo/me-benz
pyrilamine/p-eph/chlophedianol	pyrilamine/pentobarbital

pyrilamine/phenylephrine/dm	pyrilamine/phenyltolox/phenir
pyrilamine/pseudoeph/chlophed	pyrilamine/pseudoephed/carbeta
pyrilamine/pseudoephedrine/dm	ramelteon
scopolamine	scopolamine hydrobromide
scopolamine hydrochloride	scopolamine n-oxide hydrobromide
secobarbital	secobarbital sodium
suvorexant	talbutal
tasimelteon	theophylline/ephed/hydroxyzine
thiamylal sodium	xylazine hydrochloride
zolpidem tartrate	zolpidem tartrate;medical food
**Anticonvulsants**	
acetaminaphen/phenobarbital	acetazolamide
acetazolamide sodium	activated charcoal/ipecac/magnesium sulfate
aspirin /phenobarbital	aspirin/ethoheptazine citrate/meprobamate
aspirin/meprobamate	atropine sulfate/phenobarbital
benactyzine/meprobamate	biotin/cyanocobalamin/iron/magnesium sulfate/zinc
brivaracetam	calcium carbonate/phenobarbital
cannabidiol (cbd)	carbamazepine
conj. estrogens/meprobamate	conjugated estrogens/meprobamate
dehydrocholic acid/homatropine/phenobarbital	dextrose/magnesium sulfate
diclofenac na;gabapentin;lido hcl;cream, multi ing	dicylcomine hydrochloride/phenobarbital
enzymes/homatropine/phenobarbital	enzymes/phenobarbital/sodium bicarbonate
ephedrine & phenobarbital	ephedrine hydrochloride/phenobarbital/theophylline
eslicarbazepine acetate	ethosuximide
ethotoin	ethyl vanillin
ezogabine	felbamate
ferric chloride/magnesium sulfate/quinine sulfate	fosphenytoin sodium
gabapentin	gabapentin enacarbil
gabapentin/capsai/me-sal/menth	gabapentin/dietary suppl no.11
gabapentin/lidocaine	gabapentin/lidocaine/menthol
gabapentin;ketoprofen;lecithin;lido hcl;cream base	gabapentin;ketoprofen;lido hcl;cream base
gabapentin;lidocaine	gabapentin;medical food
hyoscyamine sulfate/phenobarbital	itraconazole;phenytoin sodium;cream base
lacosamide	lactated ringer s solution/magnesium sulfate
lamotrigine	levetiracetam
levetiracetam in nacl (iso-os)	magnesium sulfate
magnesium sulfate in 0.9% nacl	magnesium sulfate in water
magnesium sulfate/d5w	magnesium sulfate/polyethylene glycol
magnesium sulfate/potassium sulfate/sodium sulfate	magnesium sulfate/sodium chloride
magnesium sulfate/tta/rhubarb	mephenytoin
mephobarbital	meprobamate
meprobamate w/petn	meprobamate-ethopheptazine-asa
meprobamate/tridihexethyl	metharbital
methsuximide	oxcarbazepine
paraldehyde	paramethadione
pentaerythritol tetranitrate/phenobarbital	pentaerythritol/phenobarbital
perampanel	perampanel;perampanel

phenacemide	phenobarbital
phenobarbital sodium	phenobarbital/0.9 % sod chlor
phenobarbital/allobarbital	phenobarbital/phenytoin
phensuximide	phentermine/topiramate
phenytoin	phenytoin sodium
phenytoin sodium extended	phenytoin sodium, extended
phenytoin sodium, prompt	pregabalin
primidone	propantheline/phenobarbital
riluzole	rufinamide
stiripentol	thiopental sodium
tiagabine hcl	tiagabine hydrochloride
topiramate	trimethadione
valproate sodium	vanillin
vigabatrin	zaleplon
zonisamide	
